# Short-course performance variation across all race sections: How 100 and 200 m elite male swimmers progress between rounds

**DOI:** 10.3389/fspor.2023.1146711

**Published:** 2023-03-28

**Authors:** Francisco Cuenca-Fernández, Jesús J. Ruiz-Navarro, Marek Polach, Raúl Arellano, Dennis-Peter Born

**Affiliations:** ^1^ Aquatics Lab, Department of Physical Education and Sports, Faculty of Sport Sciences, University of Granada, Granada, Spain; ^2^ Department of Social Sciences in Kinanthropology, Palacký University Olomouc, Olomouc, Czechia; ^3^ Umimplavat.cz, Analysis and Consultation for Swimming Technique and Race Performance, Prague, Czechia; ^4^ Section for High-Performance Sports, Swiss Swimming Federation, Bern, Switzerland; ^5^ Department for Elite Sport, Swiss Federal Institute of Sport Magglingen, Magglingen, Switzerland

**Keywords:** competition analysis, pacing, race parameters, swimming, kinematic analysis

## Abstract

**Introduction:**

To investigate performance variation in all race sections, i.e., start, clean swimming, and turns, of elite short-course races for all swimming strokes and to determine the effect of performance variation on race results.

**Methods:**

Comparing finalists and non-qualified swimmers, a total of 256 races of male swimmers (*n* = 128, age: 23.3 ± 3.1, FINA points: 876 ± 38) competing in the European short-course swimming championships were analyzed. The coefficient of variation (CV) and relative change in performance (Δ%) were used to compare intra-individual performance progression between rounds and inter-individual differences between performance levels using a linear mixed model.

**Results:**

While most performance variables declined during the races (*P *< 0.005), performance was better maintained in 200 m compared to 100 m races, as well as in finalists compared to non-qualified swimmers. In 100 m races, Start Times improved between heats, semi-finals, and finals (*P *< 0.005) and contributed to the improved Split Times of Lap 1 in freestyle (*P *= 0.001, Δ = −1.09%), breaststroke (*P *< 0.001; Δ = −2.48%), and backstroke (*P *< 0.001; Δ = −1.72%). Swimmers increased stroke rate from heats/semi-finals to finals in freestyle (*P *= 0.015, Δ = 3.29%), breaststroke (*P *= 0.001, Δ = 6.91%), and backstroke (*P *= 0.005; Δ = 3.65%). Increases in stroke length and clean-swimming speed were only significant between rounds for breaststroke and backstroke (*P *< 0.005). In 200 m races, Total Time remained unchanged between rounds (*P* > 0.05), except for breaststroke (*P *= 0.008; CV = 0.7%; Δ = −0.59%). Start (*P *= 0.004; Δ = −1.72%) and Split Times (*P *= 0.009; Δ = −0.61%) only improved in butterfly. From the turn variables, OUT_5 m times improved towards the finals in breaststroke (*P *= 0.006; Δ = −1.51%) and butterfly (*P *= 0.016; Δ = −2.19%). No differences were observed for SR and SL, while clean-swimming speed improved between rounds in breaststroke only (*P *= 0.034; Δ = 0.96%).

**Discussion:**

Performance of finalists progressed between rounds in 100 m but not 200 m races, most probably due to the absence of semi-finals. Progression in 100 m races was mainly attributed to improved Start and Split Times in Lap 1, while turn performances remained unchanged. Within round comparison showed higher performance maintenance in 200 m compared to 100 m events, which showed more pronounced positive pacing. Success of finalists was attributed to their overall higher performance level and superior progression between rounds.

## 1. Introduction

As swimmers compete in their own lane and have little interference with the other competitors, performance variation is lower in swimming compared to other sports that compete in a pack (1, 2). For instance, previous studies showed that performance variation was lower in 400 m swim races compared to 1.500 m track running despite similar physiological profiles, as demonstrated by similar race times (03:45 and 03:36 mm:ss, respectively) (1). Still, pacing in swim races can change from start to finish (2), especially noticable in short course events, where the higher volume of turns can both help or hinder swimmers in developing optimal pacing strategies.

In general, highest velocities are recorded after the start during the first 15m, after which velocities continuously decrease during the clean swimming phase (3, 4). This has been characterized in the literature as an all-out pacing strategy (3, 4), and is particularly common in swimming sprint events of long- and short-course races (5). However, the pacing strategies can vary substantially on an individual basis. For instance, in races of 100 m and longer, some swimmers may choose to increase performance at the end of the race (i.e., negative pacing strategy) even after displaying a faster first lap (3). Likewise, while middle-distance races (i.e., 200 and 400 m) typically show an even and more stable pacing strategy from start to finish (3), recent research in long-course showed that 200 m events exhibited quite high variation between the four 50 m split times, presumably due to their high-intensity requirements and controlled pacing strategy (6). In this regard, it is also worth mentioning that a greater number of laps and turns increases uncertainty (7), thus providing additional opportunities to introduce performance changes. Therefore, it is reasonable to assume that these changes could also be noticeable for progressing between rounds.

Arellano and co-workers analyzed long course 50 m events and compared performance variation between heats, semi-finals, and finals. While start performance variables showed the largest performance variation, finalists’ start performance increasingly correlated with race times throughout the rounds (8). These findings are in line with a previous study in which progression from semi-finals to finals were mainly attributed to a significant improvement in the first 50 m lap time in 100 and 200 m events (9). However, to the authors’ knowledge, there are no studies that explore these underlying technical modifications when swimmers aim to progress between rounds on the short course. In addition, although athletes also change their clean swimming race strategy throughout the rounds—increasing their stroke rate (SR) and decreasing their stroke length (SL)—this did not necessarily contribute to the improved race times (8). In this sense, an interesting approach could be to analyze intra-individual variations in stroke kinematics in addition to race section times, since a clean swimming strategy (a more consistent SR, while maintaining SL) contributed to a new 100 m freestyle World Record (10).

Performance variation also differs between the 4 swimming strokes butterfly, backstroke, breaststroke, and freestyle. Firstly, butterfly and freestyle showed greater normative stability than backstroke and breaststroke (4), and secondly, male finalists’ performance progression was less pronounced between 50 m breaststroke event rounds compared to the other swimming strokes (8). Differences between swimming strokes may be related to inter-athlete performance difference (analyzed by range in FINA points achieved at the European championships), which was lower in 200 m breaststroke compared to the other swimming strokes (9). Therefore, performance variations should be analyzed not only for each race section, but also for each swimming stroke.

While the aforementioned studies analyzed long-course races, short-course races involve almost twice as many turns and different pacing strategies (11). To date only one study has focused on performance variation in short-course events (12). Cuenca-Fernandez and co-workers (12) analyzed freestyle turn performance and their sub-sections across all race distances (i.e., 50, 100, 200, 400, and 800/1,500 m) in male and female swimmers, and showed that turn times were faster in finalists and short-distance events: finalists’ turn performances were more stable than those of non-qualified swimmers and turn times were slower the longer the distance (12). Performance variation was largest prior to wall contact (IN_5 m), while wall exit variables were more consistent. The authors suggest future studies include clean swimming performance and fatigue for a more comprehensive analysis of performance variation across all race sections in short-course events (12). Analyzing performance variation of clean-swimming speed, SR, and SL in particular, may improve the understanding of pacing patterns in the various sub-sections and help elite swimmers prepare for the specific demands of short-course races.

Therefore, the aim of the study was (1) to investigate the performance variation between and within rounds in all race sections, i.e., start, clean swimming, and turn sections of elite 100 and 200 m short-course races, i.e., butterfly, backstroke, breaststroke, freestyle; and, (2) to determine the effect of performance level by comparing finalists and non-qualified swimmers, i.e., the eight fastest swimmers from the heats that did not qualify for the finals. Our hypotheses were (1) that alternations in race sections throughout the rounds will be specific to race distance, i.e., start performance mainly affecting 100 m as well as turn and clean-swimming performances 200 m races, and (2) that finalists will show more progression between rounds and more stable performances within races.

## 2. Material and methods

### 2.1. Participants

All races of male finalists (*n* = 64, age: 23.7 ± 2.8 years, FINA points: 902 ± 33, performance level 1), semi-finalists (*n* = 32, age: 23.8 ± 3.4 years, FINA points: 854 ± 19, performance level 2), and the eight fastest non-qualified swimmers from the heats (*n* = 64, age: 23.0 ± 3.4 years, FINA points: 834 ± 24, performance level 2) at the European short-course championships in Glasgow 2019 were analyzed. Performance levels were classified as suggested previously (13). To compare swimming strokes, i.e., butterfly, backstroke, breaststroke, and freestyle, only 100 and 200 m events were included in the analysis. As the 200 m short-course events are held without semi-finals, a total 128 swimmers and 224 races were analyzed. Participants were video monitored for television broadcasting and race analyses of the participating nations by the organizer of the event—Ligue Européenne de Natation (LEN). The study was preapproved by the internal review board of the Swiss Federal Institute of Sport Magglingen (Reg.-Nr. 098-LSP-191119) and is in accordance with the code of conduct of the World Medical Association for medical research involving human subjects (Declaration of Helsinki).

### 2.2. Data analysis

The swim races were video monitored by 12 cameras positioned in the mid-section of the pool, 20 m from the side of the pool and 5 m above the water surface (Spiideo, Malmö, Sweden). The cameras monitored each lane and swimmer individually with a moving view (V59 PTZ, Axis Communications AB, Lund, Sweden). Two fixed view cameras were positioned at the start and turn sections and monitored all lanes. Video footage was collected at 50 Hz. Race times were provided by the official timekeeper of the championships (Microplus Data Processing and Timing, Marene, Italy).

For the analyses, the video footage was imported to a motion analyses software (Kinovea 0.9.1; Joan Charmant & Contrib.) and synchronized to the light flash, visible at the starting signal. The analyses software allowed frame-by-frame playback and for the timecode to be saved and exported. To facilitate efficiency and accuracy, a multi-media controller was used to control playback speed and direction, and to extract timecodes (Shuttle Pro V2, Contour Design, Windham, NH, USA). The lane rope markers at 5, 10, 15, and 20 m were used for section analyses. First contact of hands (butterfly and breaststroke) or feet (backstroke and freestyle) with the wall determined the end of each lap.

The following variables were extracted from the video footage:

§ Start time: from starting signal to top of the head at the 15 m mark.
§ IN_5 m turn phase: from top of the head at 5 m before the wall to wall contact.
§ OUT_5 m turn phase: from wall contact to top of the head at 5 m after the wall.
§ OUT_5_10 m turn phase: from top of the head at 5 m after the wall to 10 m after the wall.
§ Clean-swimming speed: 15–20 m for first lap and 10–20 m for all following laps.
§ SR (Hz): dividing 60 by time of a single stroke during clean swimming phase.
§ SL (m): multiplying stroke time and section speed during clean swimming phase.

The timecode from the motion analyses software was exported to a specific Excel (Microsoft 365 Apps for enterprise, Microsoft Corporation, Redmond, WA, USA) spreadsheet to calculate the aforementioned variables and to conduct further data processing. Inter-rater reliability for this procedure has previously been described with a mean intra-class correlation coefficient of 0.98 ± 0.04 (14–16).

### 2.3. Statistical analysis

The benchmarks for the eight finalists and the eight non-qualified swimmers, were expressed for each lap and round as mean ± standard deviation. Shapiro–Wilk and Levene tests confirmed assumptions of normality and homoscedasticity, respectively. The coefficients of variation (CV) for each race-section were calculated according to Equation (1).

$$(1)\;\;\;\;\;\mathrm{Coefficcient}\;\mathrm{of}\;\mathrm{variation}\;(\mathrm{CV})\;=\;\frac{\mathrm{Standard}\;\mathrm{deviation}\;(e.g.,\;\;\mathrm{Semi}-\mathrm{final}\;\mathrm{and}\;\mathrm{Final})\;\;}{\mathrm{Mean}\;\;(e.g.,\;\;\mathrm{Semi}-\mathrm{final}\;\mathrm{and}\;\mathrm{Final})}\;\times \;100$$

As performed previously (9, 12, 17, 18), a linear mixed model was applied to estimate means for each race variable (fixed effects), with inter-individual CVs to compare performances of all swimmers in the different rounds, and with intra-individual CVs of each swimmer across the different race distances (i.e., 100 and 200 m) and levels of performance (i.e., finalists and non-finalists) as random effects (modelled as variances). The fixed main effects were the following: level of performance [e.g., finalists (*n* = 8), and non-qualified (*n* = 8)], lap number (e.g., 1^st^, 2^nd^, 3^rd^, and 4^th^ lap of a 100 m race), and race-section (i.e., Start Time, Split Times, IN_5 m, OUT_5 m, OUT_5–10 m, SR, SL and clean-swimming speed). To establish whether the intra-individual CV affects performance positively or negatively, the relative change in performance (Δ%) was obtained using Equation (2). All statistical procedures were performed using SPSS 23.0 (IBM, Chicago, IL, USA) and the level of significance was set at *P* < 0.05.

$$\begin{array}{rl} & (2)\;\mathrm{Relative}\;\mathrm{change}\;(\Delta \text{\%})\;=\\ & \;\frac{\mathrm{Performance}\;\mathrm{in}\;\mathrm{Round}\;2\;-\mathrm{Performance}\;\mathrm{in}\;\mathrm{Round}\;1\;}{\mathrm{Performance}\;\mathrm{in}\;\mathrm{Round}\;1\;}\;\times \;100 \end{array}$$

## 3. Results

Based on Total Time of the races (Table 1), performance progressed significantly through the rounds for most 100 m events (i.e., from heats to semi-finals and finals). Specifically, breaststroke displayed the largest CV and Δ% (*P* < 0.001; CV = 0.9%; Δ = −1.62%), followed by butterfly (*P* = 0.002; CV = 0.7%; Δ = −1.18%), and the smallest CV and Δ% in freestyle (*P* = 0.009; CV = 0.6%; Δ = −0.90%). Additionally, Start Times were faster during the finals compared to the heats in most strokes (Table 1). Total Time of 200 m races did not differ between rounds (*P* > 0.05), except for breaststroke (*P* = 0.008; CV = 0.7%; Δ = −0.59%), which showed faster Total Times in the finals. Over 200 m, Start Time only varied in butterfly (*P* = 0.004; Δ = −1.72%), during which it decreased in the finals.

**Table 1:** Inter-round total and start time variability obtained for the finalists (F; *n* = 8) and the non-qualified swimmers (NQ; *n* = 8) in all distances and strokes.

			Total time				Start time			
			Mean ± SD	CV	Δ%	*P*	Mean ± SD	CV	Δ%	*P*
100 m freestyle	F	H	46.78 ± 0.43	0.60	−0.90	0.009	5.59 ± 0.10	1.10	−1.26	0.054
		SF	46.51 ± 0.27				5.56 ± 0.11			
		F	46.36 ± 0.43				5.52 ± 0.13			
	NQ	H	47.43 ± 0.13	0.30	0.28	0.151	5.70 ± 0.11	1.10	−0.61	0.300
		SF	47.29 ± 0.25				5.66 ± 0.14			
100 m breaststroke	F	H	57.56 ± 0.51	0.90	−1.62	<0.001	6.38 ± 0.14	1.40	−2.48	<0.001
		SF	56.78 ± 0.46				6.26 ± 0.16			
		F	56.64 ± 0.32				6.23 ± 0.13			
	NQ	H	58.33 ± 0.32	0.70	−0.68	0.008	6.58 ± 0.17	0.70	−0.65	0.187
		SF	57.93 ± 0.22				6.53 ± 0.24			
100 m backstroke	F	H	50.71 ± 0.56	0.60	−1.01	<0.001	6.19 ± 0.10	1.10	−1.89	<0.001
		SF	50.32 ± 0.37				6.12 ± 0.11			
		F	50.20 ± 0.49				6.08 ± 0.12			
	NQ	H	51.06 ± 0.15	0.40	−0.17	0.460	6.17 ± 0.11	0.30	0.08	0.697
		SF	50.98 ± 0.37				6.17 ± 0.12			
100 m butterfly	F	H	50.43 ± 0.48	0.70	−1.18	0.002	5.62 ± 0.17	1.20	−1.48	0.012
		SF	50.13 ± 0.17				5.63 ± 0.13			
		F	49.84 ± 0.45				5.54 ± 0.12			
	NQ	H	51.09 ± 0.30	0.50	−0.66	0.003	5.70 ± 0.13	1.30	−0.88	0.220
		SF	50.76 ± 0.17				5.65 ± 0.15			
200 m freestyle	F	H	102.75 ± 0.84	0.60	−0.30	0.318	5.84 ± 0.17	1.00	−0.38	0.568
		F	102.44 ± 1.19				5.81 ± 0.20			
	NQ	H	103.91 ± 0.34				5.89 ± 0.14			
200 m breaststroke	F	H	124.09 ± 1.01	0.40	−0.59	0.008	6.57 ± 0.22	1.40	−0.22	0.796
		F	123.35 ± 0.80				6.55 ± 0.17			
	NQ	H	126.80 ± 0.90				6.84 ± 0.25			
200 m backstroke	F	H	111.01 ± 0.69	0.30	−0.06	0.733	6.41 ± 0.18	0.60	−0.23	0.615
		F	110.92 ± 1.18				6.39 ± 0.18			
	NQ	H	113.14 ± 0.58				6.56 ± 0.15			
200 m butterfly	F	H	113.05 ± 0.59	0.70	−0.73	0.052	6.08 ± 0.17	1.40	−1.72	0.004
		F	112.22 ± 1.40				5.97 ± 0.16			
	NQ	H	114.34 ± 0.43				6.03 ± 0.14			

The inter-individual linear mixed model analysis for inter-round variability including means ± standard deviation obtained for all swimming strokes, race-sections, rounds, and performance levels is presented in the supplementary material (Supplementary Table S1–S8). In regard to the 100 m races, Split Times varied significantly between rounds: changes in performance were especially pronounced in Lap 1 for freestyle (*P* = 0.001, Δ = −1.09%), breaststroke (*P* < 0.001; Δ = −2.48%), and backstroke (*P* < 0.001; Δ = −1.72%). There were no significant changes in the turn section over the rounds for any of the strokes (Supplementary Table S1–S4), although butterfly races showed significantly faster IN_5 m times (*P* = 0.006; Δ = −2.21%) from heat to semi-final. According to the mixed model analysis of clean swimming phases, swimmers increased SR from heats/semi-finals to finals in freestyle (*P* = 0.015, Δ = 3.29%), breaststroke (*P* = 0.001, Δ = 6.91%), and backstroke (*P* = 0.005; Δ = 3.65%), while only breaststroke and backstroke showed significantly increased SL and clean-swimming speed between rounds (*P* < 0.005). In the 200 m races (Supplementary Table S5–S8), Split Times only decreased significantly between rounds in breaststroke (*P* = 0.009; Δ = −0.61%), although butterfly also showed a trend (*P* = 0.051). Turn variables between rounds only varied for breaststroke (*P* = 0.006; Δ = −1.51%) and butterfly (*P* = 0.016; Δ = −2.19%), as OUT_5 m times significantly decreased during the final. Regarding the clean swimming variables, no differences were observed for SR and SL in any of the strokes over 200 m, and clean-swimming speed improved significantly between rounds only in breaststroke (*P* = 0.034; Δ = 0.96%).

**Figure 1:**
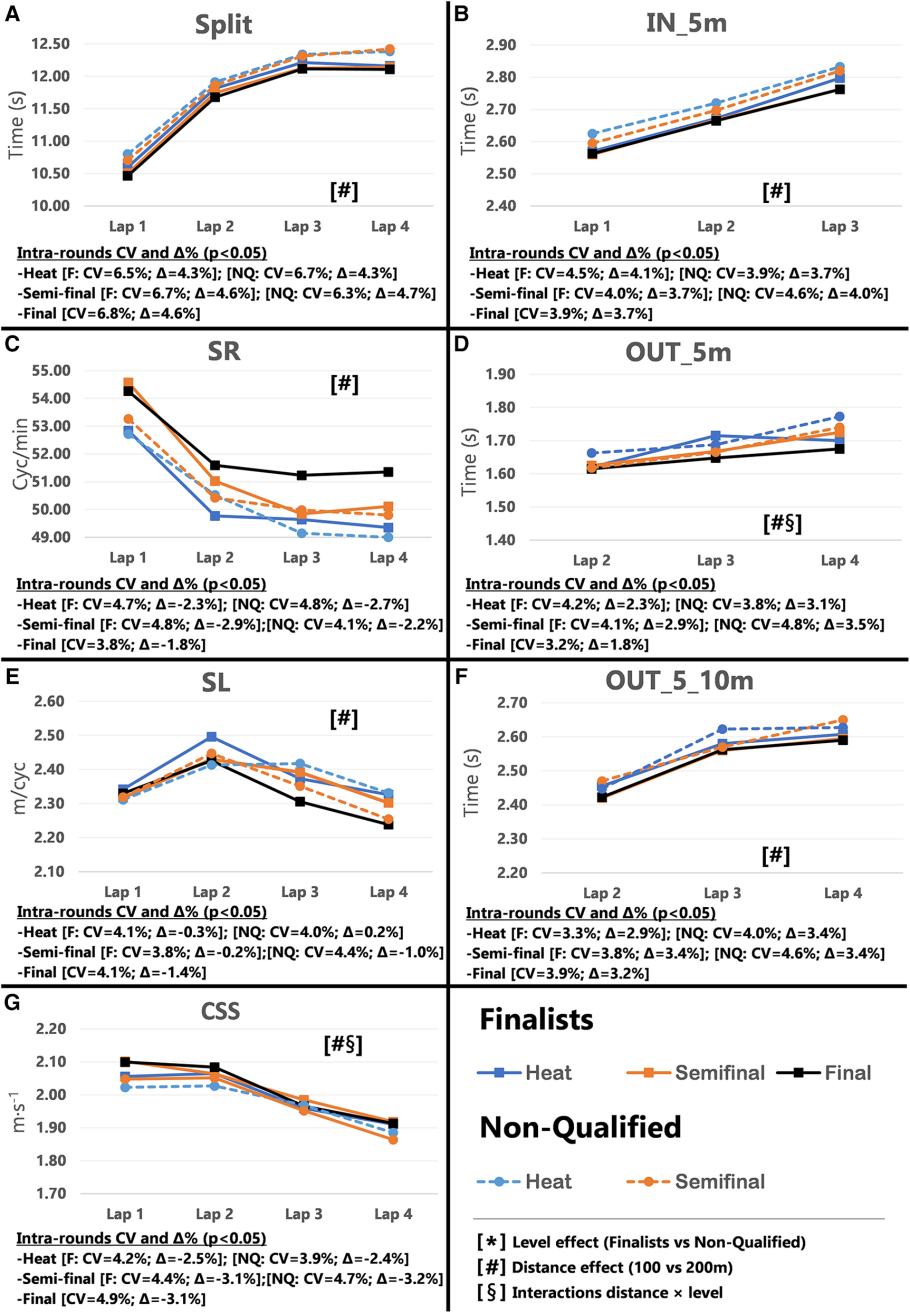
Intra-race variability in performance and random effects obtained for the finalists (*n* = 8) and the non-qualified (*n* = 8) in the 100 m freestyle. (**A**) 25 m split times, (**B**) IN_5m turn phase, (**C**) Stroke rate, (**D**) OUT_5m turn phase, (**E**) Stroke length, (**F**) OUT_5_10m turn phase, (**G**) Clean-swimming speed.

**Figure 2:**
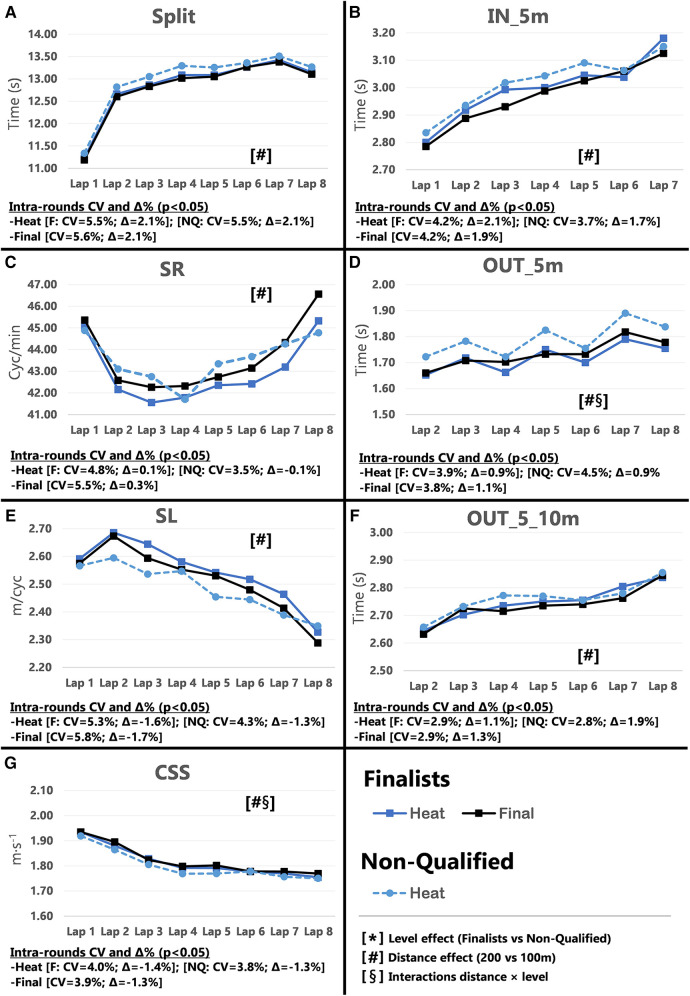
Intra-race variability in performance and random effects obtained for the finalists (*n* = 8) and the non-qualified (*n* = 8) in the 200 m freestyle. (**A**) 25 m split times, (**B**) IN_5m turn phase, (**C**) Stroke rate, (**D**) OUT_5m turn phase, (**E**) Stroke length, (**F**) OUT_5_10m turn phase, (**G**) Clean-swimming speed.

**Figure 3:**
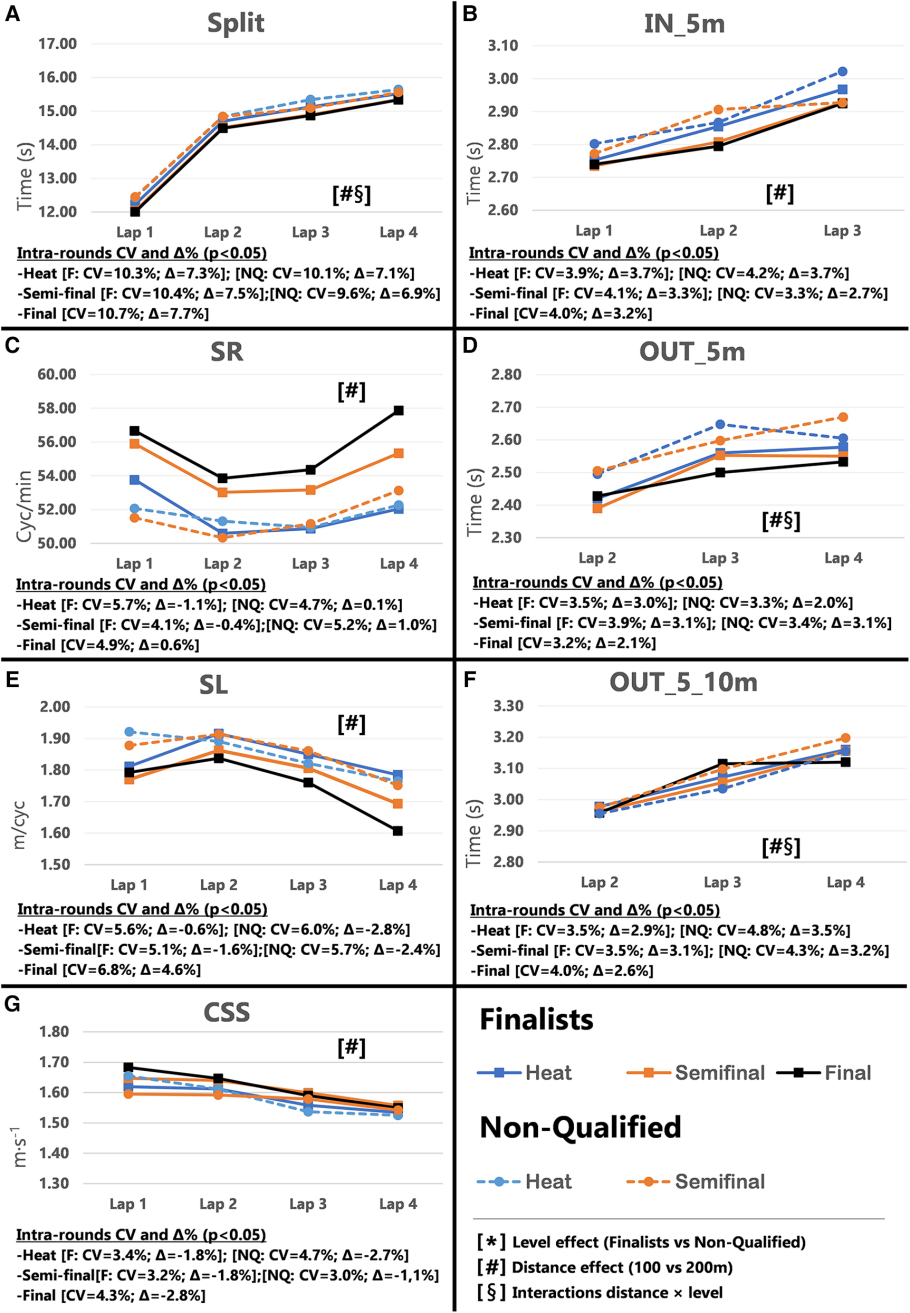
Intra-race variability in performance and random effects obtained for the finalists (*n* = 8) and the non-qualified (*n* = 8) in the 100 m breaststroke. (**A**) 25 m split times, (**B**) IN_5m turn phase, (**C**) Stroke rate, (**D**) OUT_5m turn phase, (**E**) Stroke length, (**F**) OUT_5_10m turn phase, (**G**) Clean-swimming speed.

**Figure 4:**
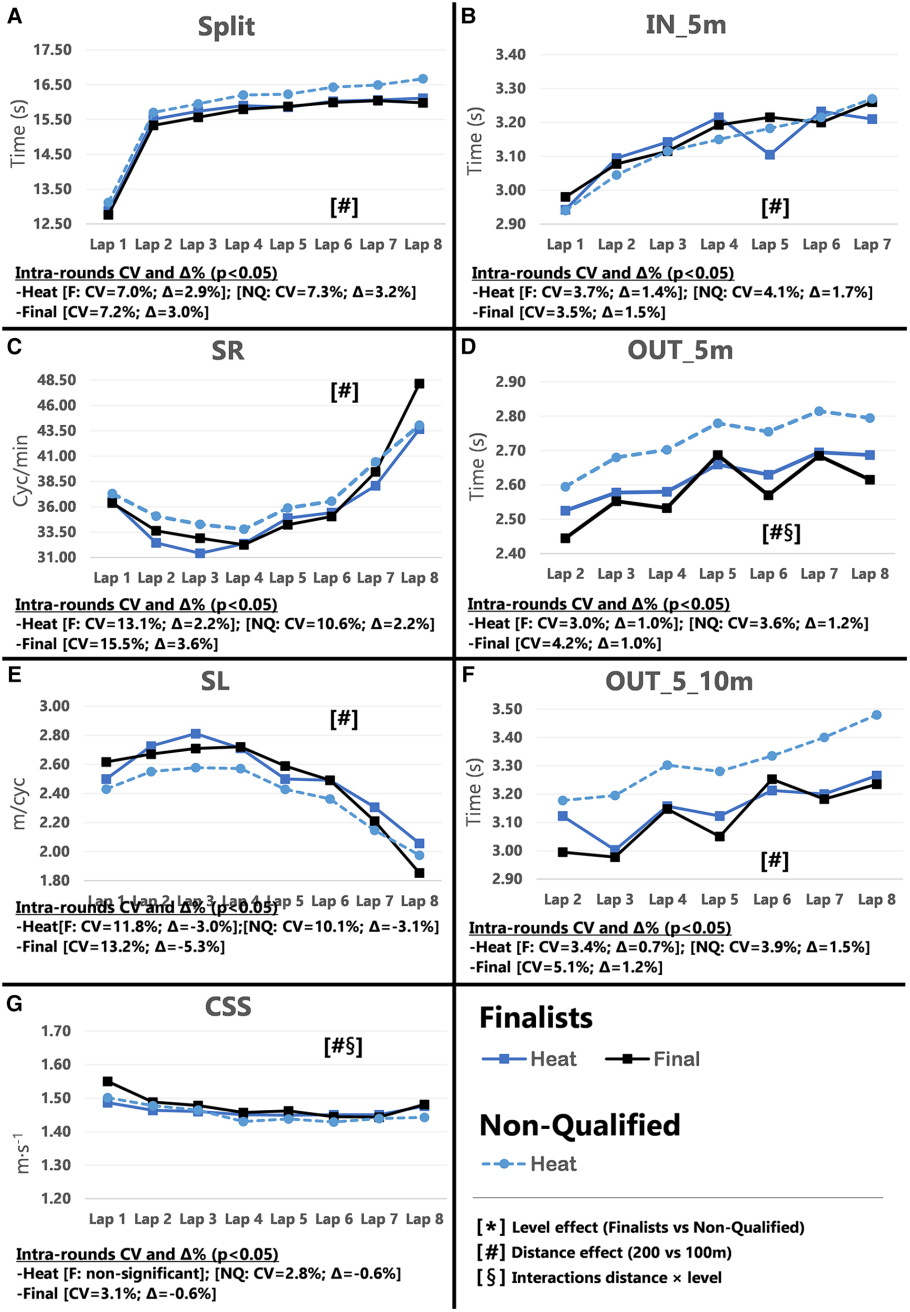
Intra-race variability in performance and random effects obtained for the finalists (*n* = 8) and the non-qualified (*n* = 8) in the 200 m breaststroke. (**A**) 25 m split times, (**B**) IN_5m turn phase, (**C**) Stroke rate, (**D**) OUT_5m turn phase, (**E**) Stroke length, (**F**) OUT_5_10m turn phase, (**G**) Clean-swimming speed.

**Figure 5:**
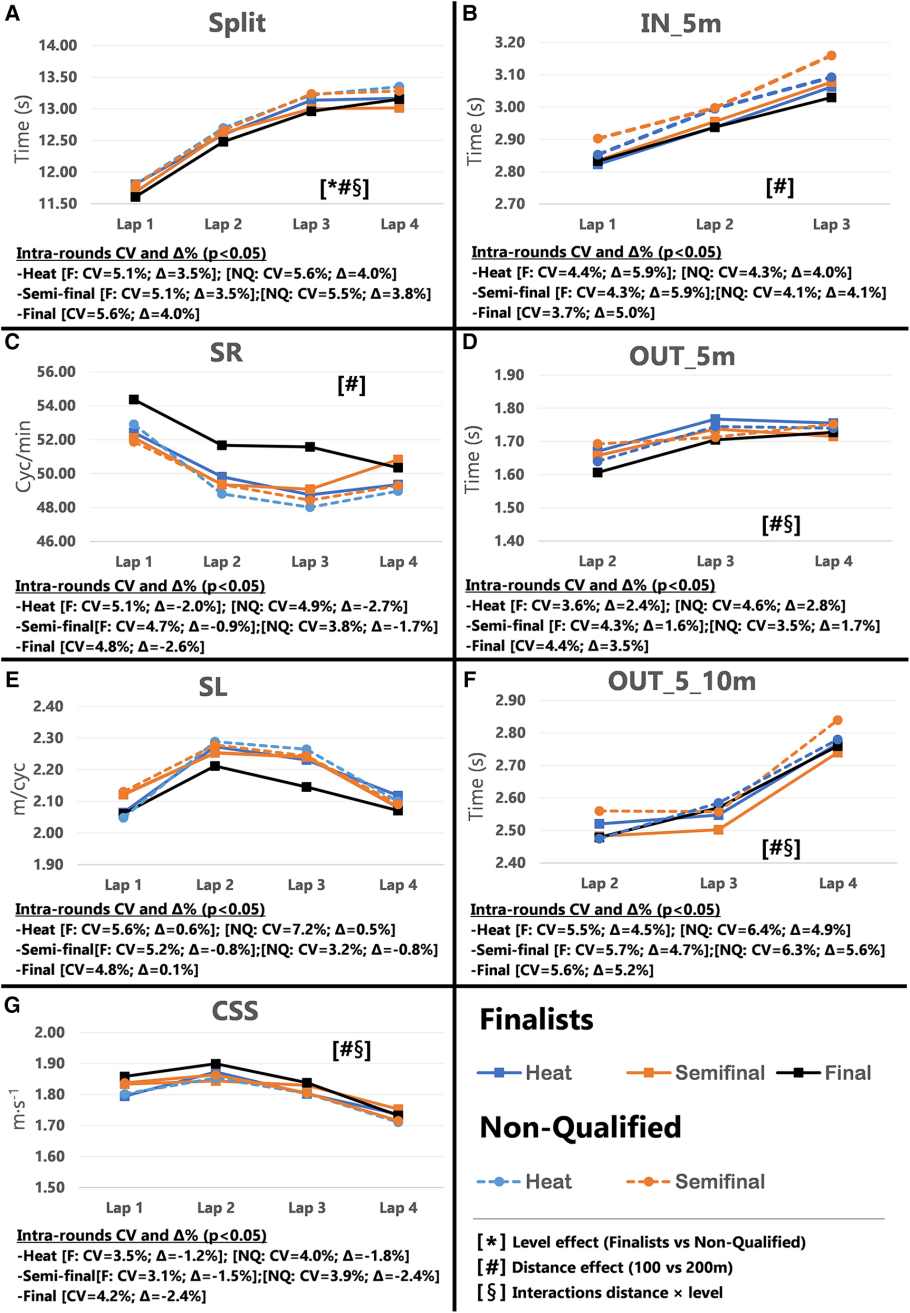
Intra-race variability in performance and random effects obtained for the finalists (*n* = 8) and the non-qualified (*n* = 8) in the 100 m backstroke. (**A**) 25 m split times, (**B**) IN_5m turn phase, (**C**) Stroke rate, (**D**) OUT_5m turn phase, (**E**) Stroke length, (**F**) OUT_5_10m turn phase, (**G**) Clean-swimming speed.

**Figure 6:**
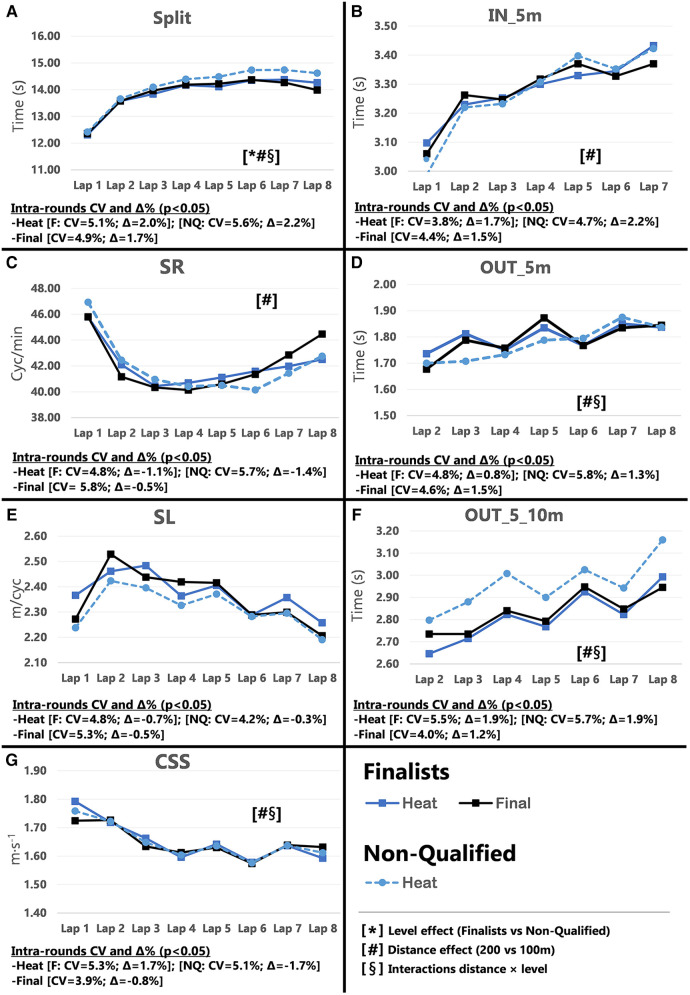
Intra-race variability in performance and random effects obtained for the finalists (*n* = 8) and the non-qualified (*n* = 8) in the 200 m backstroke. (**A**) 25 m split times, (**B**) IN_5m turn phase, (**C**) Stroke rate, (**D**) OUT_5m turn phase, (**E**) Stroke length, (**F**) OUT_5_10m turn phase, (**G**) Clean-swimming speed.

**Figure 7:**
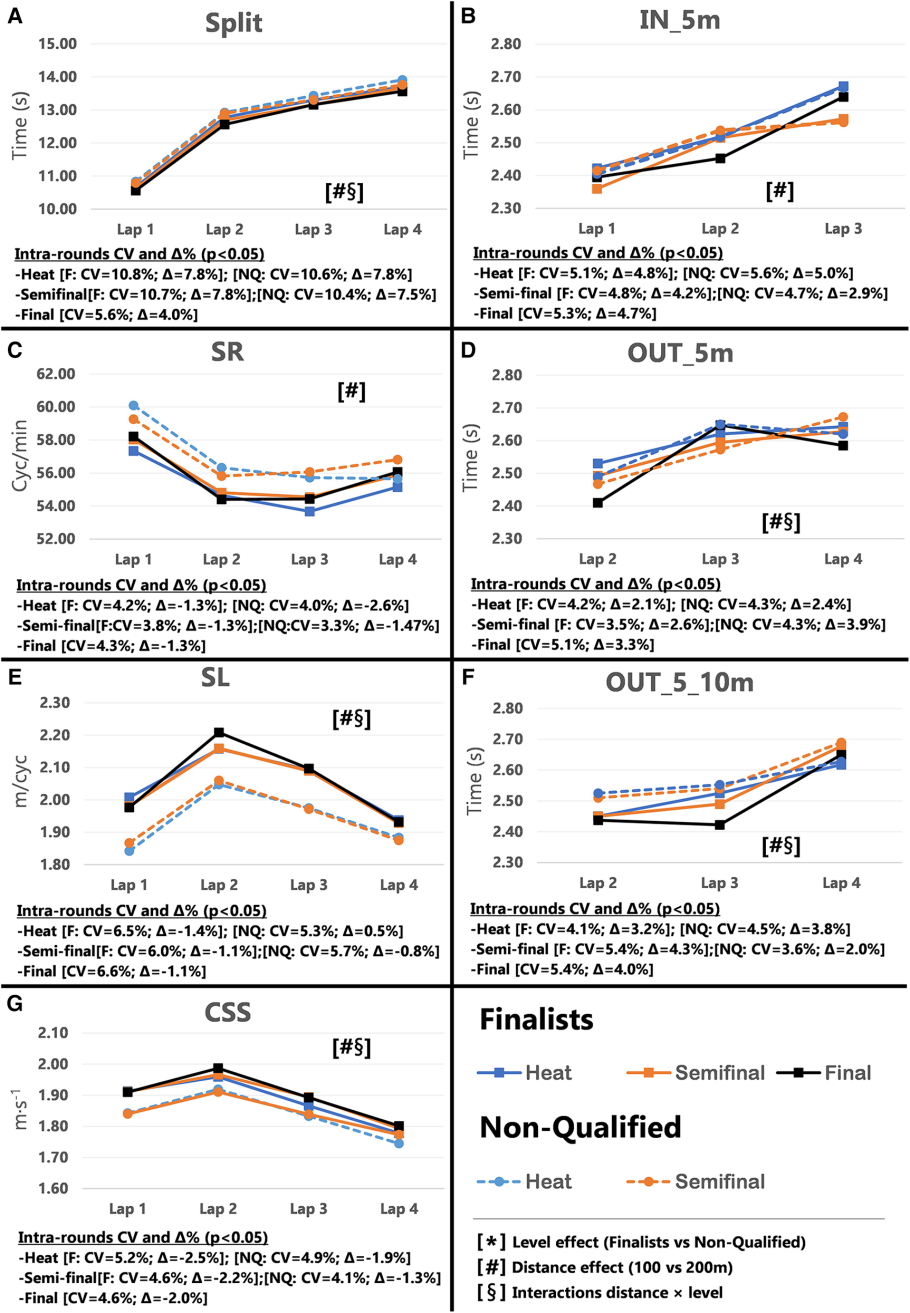
Intra-race variability in performance and random effects obtained for the finalists (*n* = 8) and the non-qualified (*n* = 8) in the 100 m butterfly. (**A**) 25 m split times, (**B**) IN_5m turn phase, (**C**) Stroke rate, (**D**) OUT_5m turn phase, (**E**) Stroke length, (**F**) OUT_5_10m turn phase, (**G**) Clean-swimming speed.

**Figure 8:**
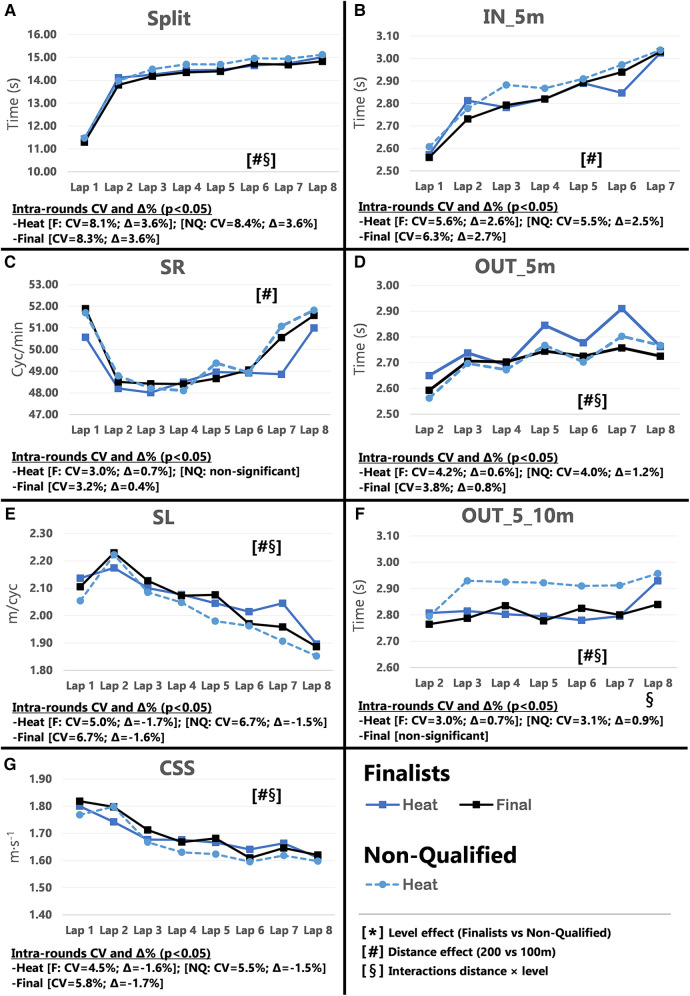
Intra-race variability in performance and random effects obtained for the finalists (*n* = 8) and the non-qualified (*n* = 8) in the 200 m butterfly. (**A**) 25 m split times, (**B**) IN_5m turn phase, (**C**) Stroke rate, (**D**) OUT_5m turn phase, (**E**) Stroke length, (**F**) OUT_5_10m turn phase, (**G**) Clean-swimming speed.

Pacing profiles and lap to lap performance variability for all distances and strokes are presented in Figures 1–8, including the CVs and Δ% values obtained from the intra-individual linear mixed effects model analyses. Performance deteriorated according to most variables, i.e., increased times as the races progressed (*P* < 0.05), with the exceptions of clean-swimming speed in 200 m breaststroke (*P* = 0.3; Figure 4) and OUT_5_10 m in 200 m butterfly (*P *= 0.5; Figure 8). Random effect obtained for distance showed larger CV and Δ% for 100 m races, while performance maintenance was better in 200 m races. Random effects obtained for performance level only showed lower CV and Δ% in the Split Times of backstroke for the finalists compared to non-qualified swimmers (Figures 5, 6). Additionally, the distance × level interactions obtained in Split Times, turn variables and clean-swimming speed also showed superior performance levels of the finalists compared to non-qualified swimmers, but a trend for larger deteriorations in performance for the finalists over distances of 100 m, and in the non-finalists, over distances of 200 m (*P* < 0.01).

## 4. Discussion

The aim of the study was to compare performance variation for all race sections between and within rounds, between all swimming strokes, and between performance levels, i.e., finalists and non-qualified swimmers. In 100 m races, finalists showed more pronounced performance improvements compared to non-qualified swimmers. Start and Split Times in Lap 1 demonstrated greatest improvements throughout the rounds. Additionally, SR increased moderately from heats to finals. In contrast, 200 m finalists showed no significant changes in performance between rounds, except for improved OUT_5 m turn sections in strokes that involve open turns (breaststroke and butterfly). With the comprehensive multi-dimensional race analyses involving all race sections across all swimming strokes, the main findings are specifically described for each swimming stroke in its particular chapter of the discussion.

### 4.1. Freestyle

The main findings for 100 m freestyle showed that a performance progression for finalists as their Total Time improved between heats and finals (*P* = 0.009; CV = 0.60%, Δ = −0.90%). However, in contrast to the hypothesis, no variations in performance were obtained in 200 m freestyle performance between heats and finals (Table 1). The absence of semi-finals in the 200 m freestyle short-course events alters pacing strategies during the heats. Even the best swimmers perform to the best of their abilities and show excellent performances in the heats to reduce the risk of not reaching the finals (9). In regard to Start Times, only 100 m finalists showed a trend towards improved performance between rounds (*P* = 0.054). Simultaneously, the inter-individual CV for Split Times decreased from the heats to the finals (*P* = 0.017, Δ = −0.92%), in particular for Lap 1 (*P *= 0.001, Δ = −1.09%). In addition to the effect of start performance on Split Times of Lap 1, previous results in short-course races have shown a close relationship between the first 25 m Split Time and Start Time (8, 19), which contributes to almost 20%–25% of the final performance of 50 m races and 8%–11% of the 100 m races (14, 20, 21). However, the percentage contribution of start performance is as low as ∼5% in 200 m races (14, 15, 22). This small contribution to Total Time may explain why some swimmers focus on other race elements when racing over 200 m. This finding is also in line with the more pronounced difference between 1^st^ and last lap in 100 m compared to 200 m races (Figures 1, 2; Plot A), which has previously been explained by a more pronounced positive pacing strategy adopted in 100 m races (4), compared to the more even pacing strategy (3) chosen for 200 m races.

Inter-individual CVs of turn section times in the 100 m races showed no significant changes in IN_5 m, OUT_5 m and OUT_5_10 m from heats to finals (refer to Supplementary Table S1). Only selected parameters revealed small changes and trends for certain laps (e.g., OUT_5 m in Lap 3 and 4), but these did not follow a consistent performance progression from heats to the finals. The 200 m races even displayed an opposite trend of impaired turn performance during the finals (e.g., OUT_5 m in Lap 6; *P* = 0.005). However, the distance × level interaction clearly revealed faster OUT_5 m times for finalists compared to non-qualified swimmers (Figures 1, 2; plot D), which confirmed their superior turn skills regardless of the ability to improve further between rounds. Therefore, the best swimmers may take more advantage from the impulse at the wall push-off, i.e., higher force production and/or better body positioning and limb alignment (12, 23) compared to the non-qualified swimmers.

During the clean swimming phase of the 100 m races, swimmers increased SR (*P* = 0.015, Δ = 3.29%) and showed a trend towards reduced SL (*P *= 0.052) when progressing from heats to finals. While this inverse relationship between SR and SL has widely been reported in the literature (24–26), it was most pronounced in Lap 4 when swimmers approach the end of the race. Due to the relationship between muscular strength and SL (27, 28), it is unclear whether changes in SR and SL over the course of the race are intentionally applied to increase clean-swimming speed, or increases in SR are used to compensate for the loss in SL as a result of fatigue. The present data hint towards the latter. For instance, the significant difference in SR and SL in Lap 4 from heats to finals was not associated with changes in clean-swimming speed. Considering the entire race, clean-swimming speed was maintained as rounds progressed, while improved Split Time of Lap 1 was associated with change of the start performance. The significant random interactions of race distance × performance level associated with the clean-swimming speed (Figures 1, 2; plot G), indicate a higher clean-swimming speed *per se* for finalists regardless of their ability to progress between rounds. While this was evident for both 100 and 200 m races, the lack of progression in SR, SL, or clean-swimming speed from heats to finals was more pronounced in the 200 m races. Furthermore, the distance effects obtained for SR, SL, and clean-swimming speed showed lower Δ% (Figures 1, 2; plots C and E), confirming that 200 m swimmers need a better control of pace and technique from first to last lap to avoid early decline in performance (3, 22).

### 4.2. Breaststroke

In 100 m races, Total Time from the heats improved throughout the rounds for both the finalists (*P* < 0.001; Δ = −1.62%) and non-qualified swimmers (*P* = 0.008; Δ = −0.68%). However, due to the overall lower performance level of the non-qualified swimmers, their improvement from heats to semi-finals was not enough to qualify them for the finals, i.e., improvements happened in a different level of performance (∼57–56 vs. ∼58–57 s). Therefore, final qualification not only requires performance progression but also a particular high performance level. In contrast to freestyle, 200 m breaststroke finalists showed a significant performance progression throughout the rounds (*P* = 0.008; Δ = −0.59%) and did not compete to the best of their capabilities during the heats. Lower inter-athlete performance difference in breaststroke—as shown by the FINA point analysis at the European swimming championships (9)—may give finalists the opportunity to go easier in the heats without too greater risk of not qualifying for the finals.

Similar to the freestyle races, 100 m finalists significantly improved their Start Time from the heats to the finals (*P* < 0.001; CV = 1.40%, Δ = −2.48%), which was also evident in the large changes of Lap 1 Split Times (refer to Supplementary Table S2). Start Times remained similar between rounds in the 200 m races (refer to discussion about freestyle). Although, analysis of 200 m Split Times revealed significant CV and Δ% and improvements in most laps, the greatest changes in performance occurred for Laps 2 & 3, but not in Lap 1, possibly because of the lack of improvements on the start. The intra-individual analysis showed distance effects in the Split Times, possibly prompted by the all-out strategy adopted in 100 m races (Δ = ∼7.33%), compared to the even pacing profile adopted in 200 m (Δ = ∼3.04%) (Figures 3, 4; Plot A).

As in the 100 m freestyle events, there were no differences in the turn variables between heats and finals, except for the distance × level interactions (*P* < 0.05), which confirmed the superior turn skills of breaststroke finalists compared to non-qualified, as hypothesized. The non-qualified swimmers’ IN_5 m performance declined from heats to semi-finals. As the IN_5 m is closely related to the clean swimming section (29, 30), it may have been affected by changes in SR and SL when approaching the wall. Interestingly, finalists’ OUT_5 m variable significantly changed from the 200 m breaststroke heats to the finals (*P* = 0.006; CV = 2.1%, Δ = −1.51%). As swimmers commonly compete in both 100 and 200 m races of the same swimming stroke, intensity of technical skills may be adapted to the length of the race. Such as, during the heats the finalists did not perform the wall push with their maximal capabilities. Once qualified for the final, they utilized their full capacity and adopted the push from the shorter 100 m races. Anyways, these results suggest that the OUT_5 m is possibly a key race element that distinguishes the best swimmers from their non-qualified peers, and confirms the importance of a good push-off from the wall and gliding abilities for success in 200 m breaststroke (21).

Similarly to freestyle racers, 100 m breaststroke swimmers increased SR (*P* = 0.001, Δ = 6.91%) and decreased SL (*P* = 0.014, Δ = −5.36%) when progressing from heats to finals. However, it should be noted that the largest change occurred from heats to semi-finals (Figure 3; plot C). Increased SR and reduced SL may have significantly improved Lap 3, although this is not necessarily the best strategy for increased speed. In contrast, no changes in SR and SL were observed between heats and the finals for the 200 m races despite significantly improved overall clean-swimming speed (*P *= 0.034; Δ = 0.96%). However, Lap 1, only showed a trend towards improved clean-swimming speed (*P *= 0.06; Δ = 4.08%), which, interestingly, coincided with the main difference in SL between heats and final (Δ = 4.47%). While overall SR was maintained from heats to finals, the distance effect in CV and Δ% from the intra-individual analysis showed an increased SR towards the end of the race, as fatigued swimmers possibly tried to increase clean-swimming speed. As in the 100 m races, the effect of the increased SR was counteracted by a progressive decline in SL (Figure 3; plots C, E and G). It should be noted that mean values across the races/rounds may conceal changes that occur within races, as highlighted by the inter-individual analysis of SL. As such, SL increased in certain laps of the finals (Lap 1 and Lap 5), but decreased in others (Lap 3 and Lap 8). While trends point towards an overall reduction in SL (*P *= 0.052), mean values of the eight laps showed similar values for heats (SL = 2.51 m/cycle) and finals (SL = 2.49 m/cycle).

### 4.3. Backstroke

As in freestyle, in the other alternating swimming stroke, only finalists’ Total Times (*P* < 0.001; CV = 0.60%; Δ = −1.01%), Start Times (*P* < 0.001; Δ = −1.89%) and Split Times (*P* = 0.012; Δ = −1.03%) improved throughout the rounds in the 100 m races. In contrast to the hypothesis, these changes were not observed over 200 m, as the absence of the semi-finals may require swimmers to perform at their best in the heats to assure qualification for the finals. Comparing Split Times of the 100 m races, finalists showed superior performance progression (i.e., from heats to semi-finals) compared to semi-finalists (Δ = −0.76 vs. Δ = −0.16%). This progression was mainly attributed to a faster Lap 1, likely influenced by the start, as discussed above (8, 31). The intra-individual change in performance as the 200 m race progressed, was probably less pronounced in the final than in the heat (Δ = 1.70% vs. Δ = 2.03%), probably because finalists increased performance at the end of the race (Figure 6; plot A).

Turn performance of the 100 m backstroke finalists was unaffected throughout the rounds. Interestingly, non-qualified swimmers showed a significant deterioration in the IN_5 m from the heats to the semi-finals—which may possibly be the reason they did not qualify for the finals (refer to Supplementary Table S3). While the random interactions of distance × level (*P* = 0.004) also confirmed the superior turning skills in 200 m backstroke finalists, turn variables did not differ between rounds in either the finalists or non-qualified swimmers. Therefore, rather than the ability to progress in performance, qualification for the final round was more a matter of performance level, as noted previously (32). Additionally, both finalists and non-qualified swimmers showed slower turn times towards the end of each race (Figure 6; Plots B, D and F), which has previously been found in freestyle races of various lengths (12) and attributed to increased fatigue throughout the race (6).

Throughout the rounds of the 100 m races, SR changed/increased the most in Lap 3 (*P* = 0.016) with similar trends for both Lap 1 and 2 (*P* = 0.06), which is in line with the improved clean-swimming speed from heats to finals (*P* = 0.012; Δ = 1.62%). This strategy appeared an effective method to modify clean-swimming speed, as there was a significant increase (*P* = 0.012; Δ = 1.62%) for the same inter-individual comparison between rounds. Nevertheless, it is worth mentioning that non-qualified swimmers showed similar values for SL and clean-swimming speed compared to the finalists in Lap 1 and 2 of the semi-finals and increase their performance from the heats to the semi-finals. However, the intra-individual analysis showed decline in SR and clean-swimming speed as the race progressed (*P* < 0.001), i.e., Lap 3 and 4, which was less pronounced in finalists (SR = −0.90%; clean-swimming speed = −1.53%) compared to non-qualified swimmers (SR = −1.76%; clean-swimming speed = −2.36%). Hence, better fatigue resistance throughout the race may provide an advantage for finalists. Clean swimming parameters generally did not change throughout the rounds of the 200 m events, but swimmers showed a more conservative pacing strategy at the beginning of the race, with an increase in pace towards the end, as demonstrated by the random distance x level interactions for clean-swimming speed (*P* < 0.001) (Figure 6; plot G). This probably illustrates a winning strategy rather than strategy to achieve a personal best time.

### 4.4. Butterfly

In 100 m butterfly, both finalists (*P* = 0.002; Δ = −1.18%) and non-qualified swimmers (*P* = 0.003; Δ = −0.66%) improved Total Time from heats to semi-/finals. Like the other simultaneous swimming stroke (breaststroke), the performance progression of the non-qualified swimmers was insufficient to qualify for the finals (Supplementary Table S4), although their performance progression from heats to semi-finals was superior to that of the finalists (Δ = −0.60% vs. Δ = −0.65%). As in the 200 m freestyle and backstroke that do not hold a semi-final, butterfly 200 m performance did not improve from the heats to the final (*P* = 0.052), as finalists probably performed to their best ability in the heats to minimize the risk missing the final.

Start Time patterns for butterfly races differed to the other swimming strokes: 100 m finalists mainly improved of the 100 m finalists from the semi-finals to the finals (*P* = 0.012; Δ = −1.48%), and only had a small effect on Lap 1 Split Times. Split Times mainly improved in Lap 2 and 3 (*P* = 0.004; Δ = −1.18%) throughout the rounds. When comparing finalists and non-qualified swimmers over the same rounds (i.e., heats to semi-finals), non-qualified swimmers’ performance changed to a greater degree (Δ = −0.60% vs. Δ = −0.65%), but occurred at a lower performance level compared to finalists (refer to Supplementary Table S4). Contrary to the other swimming strokes, Start Times in the 200 m butterfly races improved between heats and finals (*P* = 0.004; Δ = −1.72%). The trend towards improved Split Times (*P* = 0.051) in Lap 1 and 2 (Figure 8; plot A), suggests that swimmers with superior abilities in undulating kicking (33) can improve their 200 m performance from the very beginning of the race. However, the present study did not conduct kinematic and kinetic analyses of the start performances and the reason for the differences in butterfly Start Time progression compared to other swimming strokes needs further investigation.

Declines in IN_5 m turn performance of 100 m finalists, particularly during the semi-finals (*P* = 0.006; Δ = −2.21%), may have improved OUT_5 m and OUT_5_10 m times, this, however, only occurred in some laps (refer to Supplementary Table S4). The greatest changes in 200 m race turn performance were also found in the OUT_5 m, which significantly improved in the finals compared to the heats (Δ = −2.19%). Considering the characteristics of this phase (wall push off and subsequent gliding phase), it is possible that either the swimmers improved their wall push off, or did not perform at their maximum during the heats. In any case, as was obtained for the breaststroke, this race parameter (i.e., OUT_5 m) seemed to be the most modifiable by the swimmers when they wanted to vary the performance, approaching the benchmarks of the 100 m races. The random interaction by distance × level in the OUT_5_10 m variable (*P* = 0.020) indicates superior underwater skills of the finalists, which, similarly to improved start performance, may be due to superior undulating underwater kicking in this section (34).

Overall, butterfly swimmers showed similar clean swimming progression patterns to the other swimming strokes. In the 100 m races SR increased and SL decreased throughout the rounds (Figure 7; plots C and E). The 200 m clean swimming variables were unaffected and showed similar patterns between rounds. However, further analyses showed differences in specific laps. For example, during the finals, SR increased in Lap 7 (*P* = 0.023) compared to the heats, which coincided, with a decline in SL (*P* = 0.004) as seen in the other swimming strokes. Therefore, clean-swimming speed did not improve. In contrast, clean-swimming speed significantly improved in Lap 2 (*P* = 0.026), with a trend towards faster clean-swimming speed in Lap 3 (*P* = 0.06), which is in line with trends towards increased SL with maintained SR for those laps (refer to Supplementary Table S8). Additionally, the intra-individual analysis showed that swimmers maintained SR during the heats as the race progressed (*P* = 0.057), while SL diminished throughout the race in both heats and finals (*P* < 0.05). Strength skills that increase SL may be more important than SR to improve clean-swimming speed in butterfly races (27, 28, 35).

### 4.5. Pacing

As hypothesized from a previous review that swimmers may improve success chances by minimizing variation and applying an even pacing profile across all race sections could not be supported by the present data (2). In contrast, the CV and **Δ%** of intra-round variability of 100 m split times of freestyle Finalists increased from heats, to semi-finals, and to finals (*P *< 0.001). In particular, for 100 m events, a positive pacing profile with a fast initial split time was evident in the present study. Similarly, clean-swimming speed and SR were highest in the first and declined throughout the following laps. As swimmers typically start sprint races fast to avoid interference of the waves from the neighboring lanes, this strategy becomes important when competing against the strongest swimmers in the finals, hence increasing intra-round variability. As described previously, longer races, i.e., 400 and 800 m, show a more parabolic pacing strategy (2, 36). This pattern can be seen in the split times as well as the SR of the 200 m freestyle events of the present study (Figure 2; plots A and C). However, as clean-swimming speed continuously declined throughout the race, 200 m may present the intermediate distance between sprint events and the longer distances. Lower CV and **Δ%** for intra-round variability support previous findings, that better swimmer may apply a more conservative pacing strategy here. However, as discussed before, absence of semi-finals in short-course European championships may have resulted in better performance of Finalists in the heats.

### 4.6. Limitations

With the large amount of data from the comprehensive race analyses including start, turn, and clean swimming sections, the present study focused on the investigation of surface swimming parameters to allow normative comparisons with other contenders (e.g., at the same point mark), rather than providing a mechanistic and kinematic analysis. Future studies could add underwater distances and split times along with 5, 10 and 15 m times or stroke parameters as conducted before (4, 8, 32). It should be noted that the lack of performance progression could be the result of ineffective planning or the swimmers’ inability to perform at their best under the pressure at international competitions. Additionally, fatigue may affect individual performance progressions, especially as some swimmers participate in more than one race in occasion of the same championship. On the other hand, it must be recognized that tactics related to the characteristics of the swimmers and their rivals in conjunction with the distribution of lanes, i.e., swimming in lane 1 or lane 8, could explain some changes from one round to another. Actually, to progress in performance these top-elite swimmers possess unique characteristics and strategies and may have introduced specific technical or tactical modifications out of the complexity of an international swimming competitions and for reasons unknown to outside persons. Therefore, conclusions reached in this study should be interpreted under the assumption that elite sport performances are often composed by outliers.

## 5. Conclusion

The between round comparison showed that finalists progressed between rounds in 100 m races. The progression was mainly attributed to improved Start Times and Split Times in Lap 1, while turn performances remained unchanged. SR increased between rounds. However, due to the unchanged clean swimming speed (except for improvements in backstroke and breaststroke) the increased SR may be considered as a compensatory action to neutralize the loss of SL rather than an actual performance improvement. Total Time of finalists did not progress between the rounds of 200 m races except for breaststroke. Due to missing semi-finals, even the favorites may have to show best performances in the heats to assure qualification for finals. Accordingly, in 200 m races only minor alternations were detected for Start Times, Split Times, clean-swimming speed, and stroke parameters. Turn Times improved for the open turns of simultaneous swimming strokes only. The within round comparison showed higher performance maintenance in 200 m compared to 100 m events, which showed more pronounced positive pacing. Comparing performance levels, only backstroke finalists showed lower variation in Split Times compared to non-qualified swimmers. Generally, success of finalists was attributed to their overall higher performance level and superior progression between rounds.

## Data Availability

The raw data supporting the conclusions of this article will be made available by the authors, without undue reservation.
