# Supplementary material for: Short-course performance variation across all race sections: How 100 and 200 m elite male swimmers progress between rounds
Source: Front Sports Act Living. 2023 Mar 28;5:1146711. doi: 10.3389/fspor.2023.1146711 (PMC10086268; doi:10.3389/fspor.2023.1146711)
Supplement: Supplementary file 1 [file Datasheet1.pdf]

Table S1. Inter-rounds variability in performance and fixed effects obtained for the Finalists (F; n = 8) and the Non-Qualified swimmers (NQ; n = 8) in the 100m freestyle.

			**LAP 1**				**LAP 2**				**LAP 3**				**LAP 4**				**Fixed effects (H-SF-F)**			**Fixed effects (H-SF)**		
			**Mean**	**CV**	**Δ%**	**p**	**Mean**	**CV**	**Δ%**	**p**	**Mean**	**CV**	**Δ%**	**p**	**Mean**	**CV**	**Δ%**	**p**	**CV**	**Δ%**	**p**	**CV**	**Δ%**	**p**
**Split**	**F**	H	10.60 ± 0.20				11.81 ± 0.17				12.21 ± 0.11				12.16 ± 0.16									
		SF	10.50 ± 0.18	0.90	-1.09	0.001	11.74 ± 0.13	1.30	-0.89	0.199	12.12 ± 0.07	0.70	-0.76	0.033	12.14 ± 0.17	0.80	-0.29	0.599	0.90	-0.92	0.017	0.80	-0.60	0.062
		F	10.46 ± 0.20				11.67 ± 0.18				12.11 ± 0.11				12.10 ± 0.20									
	**NQ**	H	10.80 ± 0.13	0.90	-0.88	0.052	11.91 ± 0.12	0.40	-0.44	0.268	12.33 ± 0.14	0.30	-0.20	0.437	12.38 ± 0.21	0.70	0.30	0.437				0.60	-0.30	0.358
		SF	10.70 ± 0.11				11.86 ± 0.10				12.31 ± 0.11				12.42 ± 0.23									
**IN_5m**	**F**	H	2.57 ± 0.06				2.67 ± 0.10				2.79 ± 0.04													
		SF	2.55 ± 0.06	1.80	-0.29	0.619	2.66 ± 0.05	2.10	-0.28	0.976	2.73 ± 0.07	1.70	-1.26	0.250					1.90	-0.61	0.506	1.70	-0.74	0.281
		F	2.56 ± 0.11				2.66 ± 0.05				2.76 ± 0.06													
	**NQ**	H	2.62 ± 0.03	1.40	-1.15	0.112	2.72 ± 0.03	1.00	-0.83	0.155	2.83 ± 0.03	0.80	-0.44	0.440								1.10	-0.81	0.103
		SF	2.59 ± 0.06				2.69 ± 0.06				2.82 ± 0.07													
**OUT 5m**	**F**	H					1.62 ± 0.04				1.71 ± 0.08				1.70 ± 0.07									
		SF					1.62 ± 0.06	2.00	-0.30	0.874	1.66 ± 0.06	3.90	-4.09	0.121	1.64 ± 0.06	2.00	-1.49	0.017	2.60	-1.96	0.140	1.90	-0.36	0.711
		F					1.61 ± 0.07				1.64 ± 0.06				1.67 ± 0.07									
	**NQ**	H					1.66 ± 0.06	1.40	-1.15	0.051	1.69 ± 0.03	1.00	-0.83	0.529	1.75 ± 0.04	0.80	-0.44	0.114				2.40	-2.00	0.081
		SF					1.62 ± 0.04				1.66 ± 0.11				1.74 ± 0.05									
**OUT 5_10m**	**F**	H					2.45 ± 0.03				2.58 ± 0.03				2.60 ± 0.04									
		SF					2.42 ± 0.05	1.40	-1.34	0.220	2.56 ± 0.06	1.60	-0.68	0.029	2.59 ± 0.08	1.80	-0.67	0.310	1.60	-0.90	0.159	1.56	-0.90	0.096
		F					2.42 ± 0.02				2.56 ± 0.06				2.59 ± 0.05									
	**NQ**	H					2.47 ± 0.03	9.99	-0.91	0.006	2.57 ± 0.05	9.99	2.01	0.273	2.65 ± 0.08	9.99	-0.85	0.092				1.80	0.07	0.890
		SF					2.44 ± 0.05				2.62 ± 0.13				2.62 ± 0.05									
**SR**	**F**	H	52.83 ± 3.39				49.76 ± 3.18				49.63 ± 2.89				49.35 ± 1.97									
		SF	54.57 ± 3.19	2.70	2.62	0.068	51.02 ± 2.07	3.30	3.53	0.091	49.84 ± 1.66	3.10	3.11	0.112	50.11 ± 2.52	2.60	3.89	0.004	2.90	3.29	0.015	2.60	1.89	0.110
		F	54.26 ± 3.67				51.59 ± 2.36				51.23 ± 1.87				51.35 ± 2.03									
	**NQ**	H	52.72 ± 3.54	2.50	1.02	0.362	50.52 ± 2.45	1.70	-0.19	0.800	49.14 ± 3.81	2.80	1.68	0.159	48.70 ± 3.54	1.70	2.19	0.079				2.20	1.17	0.121
		SF	53.26 ± 4.27				50.42 ± 2.55				49.98 ± 3.32				49.80 ± 3.43									
**SL**	**F**	H	2.34 ± 0.12				2.49 ± 0.14				2.37 ± 0.09				2.32 ± 0.10									
		SF	2.31 ± 0.13	2.70	-0.49	0.436	2.43 ± 0.11	2.40	-2.88	0.042	2.39 ± 0.11	3.40	-2.93	0.111	2.30 ± 0.11	2.40	-3.92	0.004	2.70	-2.55	0.052	1.80	-1.01	0.288
		F	2.33 ± 0.15				2.42 ± 0.08				2.30 ± 0.09				2.23 ± 0.09									
	**NQ**	H	2.31 ± 0.15	0.50	0.31	0.690	2.41 ± 0.14	2.8	1.38	0.206	2.41 ± 0.21	3.4	-2.82	0.138	2.33 ± 0.14	2.6	-3.4	0.043				2.30	-1.13	0.208
		SF	2.31 ± 0.17				2.44 ± 0.13				2.35 ± 0.14				2.25 ± 0.14									
**CSS**	**F**	H	2.05 ± 0.05				2.06 ± 0.07				1.95 ± 0.05				1.91 ± 0.02									
		SF	2.10 ± 0.04	1.60	2.07	0.036	2.06 ± 0.04	2.20	0.93	0.658	1.98 ± 0.06	1.60	0.34	0.324	1.91 ± 0.03	0.70	0.14	0.565	1.50	0.87	0.119	0.80	0.09	0.065
		F	2.09 ± 0.05				2.08 ± 0.07				1.96 ± 0.03				1.91 ± 0.04									
	**NQ**	H	2.02 ± 0.04	2.30	1.21	0.044	2.02 ± 0.04	1.00	1.18	0.204	1.97 ± 0.05	0.60	-0.87	0.302	1.88 ± 0.04	0.90	-1.12	0.214				1.20	0.09	0.788
		SF	2.04 ± 0.04				2.05 ± 0.03				1.95 ± 0.03				1.86 ± 0.05									

Table S2. Inter-rounds variability in performance and fixed effects obtained for the Finalists (F; n = 8) and the Non-Qualified swimmers (NQ; n = 8) in the 100m breaststroke.

			LAP 1				LAP 2				LAP 3				LAP 4				Fixed effects (H-SF-F)			Fixed effects (H-SF)		
			**Mean**	**CV**	**Δ%**	**p**	**Mean**	**CV**	**Δ%**	**p**	**Mean**	**CV**	**Δ%**	**p**	**Mean**	**CV**	**Δ%**	**p**	**CV**	**Δ%**	**p**	**CV**	**Δ%**	**p**
**Split**	**F**	H	12.22 ± 0.17				14.69 ± 0.17				15.12 ± 0.19				15.51 ± 0.20									
		SF	12.04 ± 0.19	1.37	-2.36	0.001	14.51 ± 0.14	0.90	-1.41	0.001	14.89 ± 0.21	1.19	-1.71	0.003	15.34 ± 0.22	1.16	-1.19	0.052	1.15	-1.67	0.001	1.38	-1.38	0.004
		F	11.94 ± 0.12				14.49 ± 0.17				14.87 ± 0.17				15.33 ± 0.13									
	**NQ**	H	12.42 ± 0.21	1.38	0.16	0.838	14.83 ± 0.14	0.09	-1.19	1.0	15.34 ± 0.14	2.03	-1.63	0.001	15.64 ± 0.12	0.81	-0.54	0.357				1.08	-0.50	0.059
		SF	12.44 ± 0.22				14.83 ± 0.15				15.09 ± 0.07				15.55 ± 0.24									
**IN_5m**	**F**	H	2.75 ± 0.08				2.86 ± 0.06				2.97 ± 0.13													
		SF	2.74 ± 0.06	1.95	-0.45	0.844	2.81 ± 0.08	2.60	-2.14	0.227	2.93 ± 0.07	2.16	-1.45	0.381					2.23	-1.35	0.153	2.23	-1.23	0.173
		F	2.74 ± 0.08				2.80 ± 0.08				2.92 ± 0.04													
	**NQ**	H	2.80 ± 0.05	1.94	-1.08	0.390	2.86 ± 0.11	1.75	1.33	0.211	3.02 ± 0.08	2.40	-3.24	0.009								2.03	-0.99	0.107
		SF	2.77 ± 0.13				2.90 ± 0.10				2.92 ± 0.12													
**OUT 5m**	**F**	H					2.42 ± 0.06				2.56 ± 0.72				2.57 ± 0.07									
		SF					2.39 ± 0.09	1.81	0.30	0.342	2.55 ± 0.07	2.96	-2.40	0.268	2.55 ± 0.03	1.92	-1.77	0.190	2.23	-1.28	0.265	2.02	-0.87	0.398
		F					2.42 ± 0.04				2.50 ± 0.10				2.53 ± 0.03									
	**NQ**	H					2.49 ± 0.09	1.55	-1.15	0.650	2.64 ± 0.12	2.13	-0.83	0.176	2.60 ± 0.13	2.67	-0.44	0.106				2.40	-2.00	0.736
		SF					2.50 ± 0.08				2.59 ± 0.06				2.67 ± 0.10									
**OUT 5_10m**	**F**	H					2.97 ± 0.09				3.07 ± 0.17				3.16 ± 0.03									
		SF					2.96 ± 0.07	2.28	-0.67	0.875	3.05 ± 0.08	2.99	1.36	0.436	3.15 ± 0.05	1.24	-1.28	0.082	2.17	-0.19	0.865	1.74	-0.41	0.628
		F					2.95 ± 0.10				3.11 ± 0.13				3.12 ± 0.07									
	**NQ**	H					2.97 ± 0.10	2.02	-0.67	0.662	3.09 ± 0.20	1.75	-2.05	0.035	3.19 ± 0.16	1.82	-1.34	0.239				1.86	-1.36	0.180
		SF					2.95 ± 3.03				3.03 ± 0.25				3.15 ± 0.15									
**SR**	**F**	H	53.77 ± 3.44				50.59 ± 3.14				50.88 ± 4.45				52.04 ± 5.47									
		SF	55.90 ± 2.36	5.01	5.11	0.128	53.02 ± 3.09	5.16	6.05	0.044	53.17 ± 2.13	4.42	6.41	0.003	55.34 ± 3.30	5.74	3.89	0.001	5.08	6.91	0.001	4.25	4.66	0.018
		F	56.66 ± 4.50				53.85 ± 3.21				54.36 ± 3.43				57.87 ± 1.63									
	**NQ**	H	52.07 ± 4.53	1.17	-1.06	0.780	51.31 ± 4.55	2.75	-1.95	0.592	50.93 ± 4.20	1.09	0.43	0.644	52.26 ± 4.99	3.99	1.62	0.405				2.25	-0.23	0.920
		SF	51.52 ± 6.13				50.33 ± 5.42				51.16 ± 4.27				53.13 ± 3.90									
**SL**	**F**	H	1.81 ± 0.08				1.91 ± 0.08				1.85 ± 0.16				1.78 ± 0.17									
		SF	1.76 ± 0.06	4.79	-1.07	0.700	1.86 ± 0.13	4.97	-4.25	0.267	1.80 ± 0.06	4.05	-5.10	0.092	1.69 ± 0.09	5.32	-11.02	0.001	4.78	-5.36	0.014	3.31	-3.25	0.101
		F	1.79 ± 0.16				1.83 ± 0.07				1.76 ± 0.10				1.60 ± 0.04									
	**NQ**	H	1.92 ± 0.27	2.48	-2.28	0.614	1.89 ± 0.11	3.27	1.18	0.763	1.82 ± 0.16	2.54	2.11	0.238	1.76 ± 0.17	3.38	-0.76	0.689				2.92	0.06	0.977
		SF	1.87 ± 0.20				1.91 ± 0.19				1.86 ± 0.13				1.75 ± 0.15									
**CSS**	**F**	H	1.61 ± 0.05				1.61 ± 0.06				1.55 ± 0.03				1.53 ± 0.03									
		SF	1.64 ± 0.04	2.71	3.76	0.049	1.64 ± 0.03	2.81	2.10	0.287	1.59 ± 0.04	1.95	1.99	0.050	1.55 ± 0.04	1.32	0.97	0.069	2.20	2.21	0.001	3.01	1.85	0.001
		F	1.68 ± 0.06				1.64 ± 0.07				1.59 ± 0.04				1.54 ± 0.01									
	**NQ**	H	1.65 ± 0.17	1.32	-3.75	0.269	1.61 ± 0.09	1.14	-1.21	0.384	1.53 ± 0.06	2.58	2.62	0.026	1.52 ± 0.02	1.17	1.10	0.128				1.55	-0.30	0.778
		SF	1.59 ± 0.05				1.59 ± 0.09				1.57 ± 0.05				1.54 ± 0.03									

Table S3. Inter-rounds variability in performance and fixed effects obtained for the Finalists (F; n = 8) and the Non-Qualified swimmers (NQ; n = 8) in the 100m backstroke.

			LAP 1				LAP 2				LAP 3				LAP 4				Fixed effects (H-SF-F)			Fixed effects (H-SF)		
			Mean	CV	Δ%	p	Mean	CV	Δ%	p	Mean	CV	Δ%	p	Mean	CV	Δ%	p	CV	Δ%	p	CV	Δ%	p
**Split**	**F**	H	11.80 ± 0.08				12.59 ± 0.11				13.13 ± 0.22				13.16 ± 0.36									
		SF	11.68 ± 0.15	1.01	-1.72	0.001	12.62 ± 0.11	0.97	-0.92	0.050	13.00 ± 0.22	0.89	-1.36	0.005	13.01 ± 0.22	1.68	-0.11	0.336	1.14	-1.03	0.012	1.02	-0.76	0.036
		F	11.60 ± 0.16				12.48 ± 0.13				12.93 ± 0.20				13.15 ± 0.30									
	**NQ**	H	11.80 ± 0.14	0.45	0.00	1.0	12.69 ± 0.13	0.29	-0.25	0.126	13.22 ± 0.15	0.72	0.11	0.797	13.31 ± 0.19	0.86	-0.50	0.364				0.58	-0.16	0.542
		SF	11.80 ± 0.11				12.66 ± 0.09				13.23 ± 0.13				13.28 ± 0.22									
**IN_5m**	**F**	H	2.82 ± 0.08				2.93 ± 0.05				3.06 ± 0.08													
		SF	2.83 ± 0.08	1.51	0.35	0.885	2.95 ± 0.08	1.59	0.01	0.729	3.07 ± 0.09	1.56	-1.07	0.166					1.55	0.50	0.405	2.23	0.50	0.379
		F	2.83 ± 0.09				2.93 ± 0.03				3.03 ± 0.11													
	**NQ**	H	2.85 ± 0.07	1.72	-1.08	0.004	2.99 ± 0.06	0.08	1.33	0.866	3.09 ± 0.08	2.13	-3.24	0.036								1.32	1.31	0.034
		SF	2.90 ± 0.09				2.99 ± 0.08				3.16 ± 0.09													
**OUT 5m**	**F**	H					1.67 ± 0.05				1.76 ± 0.07				1.75 ± 0.09									
		SF					1.65 ± 0.08	3.46	-3.96	0.160	1.73 ± 0.12	3.47	-3.66	0.158	1.71 ± 0.04	3.09	-1.59	0.400	3.34	-3.07	0.088	2.95	-1.60	0.262
		F					1.60 ± 0.07				1.70 ± 0.04				1.72 ± 0.06									
	**NQ**	H					1.64 ± 0.09	3.04	3.10	0.146	1.74 ± 0.07	2.53	-1.89	0.287	1.74 ± 0.09	3.49	0.71	0.744				3.02	0.63	0.569
		SF					1.69 ± 0.10				1.71 ± 0.07				1.75 ± 0.10									
**OUT 5_10m**	**F**	H					2.52 ± 0.06				2.54 ± 0.08				2.76 ± 0.09									
		SF					2.48 ± 0.07	2.55	-1.66	0.424	2.50 ± 0.08	2.37	0.87	0.130	2.74 ± 0.09	2.39	-0.27	0.716	2.44	-0.35	0.245	1.87	-1.43	0.091
		F					2.47 ± 0.08				2.57 ± 0.07				2.76 ± 0.09									
	**NQ**	H					2.56 ± 0.10	3.22	-3.43	0.072	2.55 ± 0.08	3.23	1.06	0.583	2.84 ± 0.05	2.16	-2.15	0.070				2.87	-1.52	0.100
		SF					2.47 ± 0.11				2.58 ± 0.15				2.78 ± 0.09									
**SR**	**F**	H	52.42 ± 4.47				49.81 ± 4.73				48.75 ± 4.43				49.34 ± 2.97									
		SF	52.11 ± 3.91	2.97	3.57	0.061	49.35 ± 3.77	3.87	3.59	0.058	49.07 ± 2.32	4.07	5.46	0.016	50.83 ± 4.34	2.86	1.99	0.304	3.44	3.65	0.005	2.21	0.05	0.611
		F	54.36 ± 4.35				51.67 ± 3.16				51.56 ± 3.18				50.34 ± 2.83									
	**NQ**	H	52.90 ± 3.09	1.69	-1.98	0.067	48.80 ± 3.41	1.85	1.14	0.275	48.01 ± 3.27	1.26	0.85	0.285	48.97 ± 2.20	1.59	0.65	0.544				1.60	0.16	0.828
		SF	51.87 ± 6.13				49.36 ± 5.42				48.43 ± 4.27				49.29 ± 3.90									
**SL**	**F**	H	2.06 ± 0.13				2.27 ± 0.21				2.23 ± 0.16				2.11 ± 0.13									
		SF	2.12 ± 0.15	2.70	-0.17	0.056	2.25 ± 0.17	2.26	-2.75	0.248	2.24 ± 0.09	3.33	-3.97	0.047	2.08 ± 0.16	2.33	-2.28	0.148	2.66	-2.29	0.041	1.92	0.13	0.92
		F	2.06 ± 0.15				2.21 ± 0.12				2.14 ± 0.13				2.07 ± 0.13									
	**NQ**	H	2.04 ± 0.10	3.2	3.85	0.028	2.28 ± 0.17	1.52	-0.45	0.597	2.26 ± 0.18	1.89	-0.94	0.351	2.09 ± 0.09	1.51	-0.42	0.616				2.03	0.51	0.525
		SF	2.12 ± 0.10				2.27 ± 0.20				2.24 ± 0.15				2.09 ± 0.07									
**CSS**	**F**	H	1.79 ± 0.04				1.87 ± 0.04				1.80 ± 0.05				1.73 ± 0.05									
		SF	1.83 ± 0.03	1.18	3.40	0.001	1.84 ± 0.03	2.13	1.40	0.030	1.83 ± 0.06	2.11	1.94	0.133	1.75 ± 0.03	2.63	-0.22	0.377	2.01	1.63	0.012	1.60	0.74	0.172
		F	1.85 ± 0.04				1.89 ± 0.04				1.83 ± 0.04				1.73 ± 0.05									
	**NQ**	H	1.80 ± 0.06	1.65	1.91	0.040	1.85 ± 0.03	0.92	0.54	0.326	1.80 ± 0.05	1.28	0.06	0.930	1.71 ± 0.02	1.17	0.26	0.733				1.25	0.69	0.182
		SF	1.83 ± 0.04				1.86 ± 0.03				1.80 ± 0.03				1.71 ± 0.04									

Table S4. Inter-rounds variability in performance and fixed effects obtained for the Finalists (F; n = 8) and the non-qualified swimmers (NQ; n = 8) in the 100m butterfly.

			LAP 1				LAP 2				LAP 3				LAP 4				Fixed effects (H-SF-F)			Fixed effects (H-SF)		
			Mean	CV	Δ%	p	Mean	CV	Δ%	p	Mean	CV	Δ%	p	Mean	CV	Δ%	p	CV	Δ%	p	CV	Δ%	p
**Split**	F	H	10.66 ± 0.23				12.77 ± 0.23				13.29 ± 0.16				13.69 ± 0.27									
		SF	10.61 ± 0.16	0.83	-0.99	0.117	12.68 ± 0.09	1.21	-1.71	0.022	13.15 ± 0.12	0.95	-1.04	0.058	13.67 ± 0.15	1.01	-0.97	0.189	1.00	-1.18	0.004	0.86	-0.60	0.081
		F	10.56 ± 0.14				12.56 ± 0.14				13.16 ± 0.15				13.56 ± 0.24									
	NQ	H	10.83 ± 0.19	0.82	-0.39	0.458	12.92 ± 0.14	0.78	-0.17	0.700	13.43 ± 0.17	0.90	-0.97	0.063	13.91 ± 0.18	1.06	-1.07	0.045				0.89	-0.65	0.025
		SF	10.79 ± 0.13				12.90 ± 0.12				13.30 ± 0.19				13.76 ± 0.09									
**IN_5m**	F	H	2.42 ± 0.05				2.51 ± 0.07				2.67 ± 0.10													
		SF	2.36 ± 0.11	2.76	-1.14	0.182	2.51 ± 0.09	2.13	-2.65	0.013	2.57 ± 0.07	2.99	-1.23	0.039					2.63	-1.67	0.043	2.16	-2.21	0.006
		F	2.39 ± 0.11				2.45 ± 0.07				2.64 ± 0.08													
	NQ	H	2.40 ± 0.09	2.96	0.41	0.821	2.51 ± 0.05	2.09	0.78	0.496	2.66 ± 0.08	3.83	-4.09	0.042								2.96	-0.95	0.303
		SF	2.41 ± 0.09				2.53 ± 0.07				2.56 ± 0.11													
**OUT 5m**	F	H					2.53 ± 0.09				2.62 ± 0.08				2.64 ± 0.17									
		SF					2.49 ± 0.07	3.43	-4.97	0.013	2.59 ± 0.10	3.37	1.03	0.511	2.62 ± 0.07	2.85	-2.22	0.321	3.21	-2.05	0.118	2.84	-1.01	0.301
		F					2.41 ± 0.08				2.64 ± 0.12				2.58 ± 0.11									
	NQ	H					2.49 ± 0.09	2.49	-0.91	0.560	2.65 ± 0.12	3.70	-3.01	0.117	2.62 ± 0.08	2.84	1.96	0.355				3.01	-0.65	0.650
		SF					2.46 ± 0.09				2.57 ± 0.07				2.67 ± 0.14									
**OUT 5_10m**	F	H					2.45 ± 0.09				2.52 ± 0.11				2.61 ± 0.09									
		SF					2.45 ± 0.07	2.33	-0.51	0.887	2.49 ± 0.14	2.82	-4.23	0.009	2.67 ± 0.06	1.84	1.22	0.047	2.33	-1.17	0.143	1.97	0.27	0.712
		F					2.43 ± 0.07				2.42 ± 0.11				2.65 ± 0.09									
	NQ	H					2.51 ± 0.09	1.41	0.59	0.535	2.54 ± 0.14	1.91	0.48	0.643	2.69 ± 0.10	2.33	-2.37	0.054				1.88	-0.43	0.597
		SF					2.52 ± 0.07				2.55 ± 0.13				2.62 ± 0.09									
**SR**	F	H	57.34 ± 3.66				54.65 ± 2.59				53.67 ± 3.65				55.14 ± 2.56									
		SF	58.03 ± 3.75	2.38	1.50	0.489	54.81 ± 2.98	3.10	-0.45	0.940	54.54 ± 2.79	2.79	1.39	0.538	55.87 ± 1.99	2.70	1.64	0.478	2.74	1.02	0.603	1.95	1.09	0.172
		F	58.21 ± 4.17				54.40 ± 5.22				54.43 ± 3.91				56.07 ± 2.87									
	NQ	H	60.09 ± 2.66	2.46	-1.41	0.366	56.31 ± 2.63	1.32	-0.89	0.317	55.72 ± 1.56	1.63	0.61	0.543	55.65 ± 2.47	2.14	2.03	0.082				1.89	0.08	0.905
		SF	59.26 ± 3.20				55.81 ± 2.83				56.07 ± 2.76				56.80 ± 2.15									
**SL**	F	H	2.00 ± 0.12				2.15 ± 0.16				2.09 ± 0.14				1.93 ± 0.07									
		SF	1.98 ± 0.13	3.39	-1.56	0.739	2.15 ± 0.14	3.46	2.29	0.490	2.08 ± 0.13	2.69	0.16	0.982	1.92 ± 0.08	2.59	-0.30	0.953	3.03	0.14	0.866	2.36	-0.47	0.668
		F	1.97 ± 0.15				2.20 ± 0.21				2.09 ± 0.17				1.93 ± 0.09									
	NQ	H	1.84 ± 0.11	4.55	1.35	0.622	2.04 ± 0.09	2.09	0.59	0.648	1.97 ± 0.06	2.21	-0.15	0.911	1.88 ± 0.07	1.41	-0.44	0.591				2.57	0.33	0.716
		SF	1.86 ± 0.10				2.05 ± 0.14				1.97 ± 0.10				1.87 ± 0.07									
**CSS**	F	H	1.91 ± 0.08				1.95 ± 0.08				1.86 ± 0.07				1.77 ± 0.03									
		SF	1.91 ± 0.10	3.26	-0.18	0.995	1.96 ± 0.04	2.45	1.38	0.550	1.89 ± 0.04	1.82	1.39	0.229	1.79 ± 0.02	1.13	1.29	0.105	2.17	0.97	0.447	2.03	0.65	0.421
		F	1.90 ± 0.08				1.98 ± 0.06				1.89 ± 0.03				1.80 ± 0.04									
	NQ	H	1.84 ± 0.10	2.82	-0.11	0.957	1.91 ± 0.06	1.94	-0.44	0.708	1.83 ± 0.03	1.36	0.33	0.717	1.74 ± 0.02	1.44	1.61	0.094				1.89	0.34	0.663
		SF	1.84 ± 0.06				1.91 ± 0.05				1.83 ± 0.06				1.77 ± 0.03									

Table S5. Inter-rounds variability in performance and fixed effects obtained for the Finalists (n = 8) and the Non-Qualified swimmers (n = 8) in the 200m freestyle.

			LAP 1				LAP 2				LAP 3				LAP 4				Fixed effects (H-F)		
			**Mean**	**CV**	**Δ%**	**p**	**Mean**	**CV**	**Δ%**	**p**	**Mean**	**CV**	**Δ%**	**p**	**Mean**	**CV**	**Δ%**	**p**	**CV**	**Δ%**	**p**
**Split**	**F**	H	11.22 ± 0.18	0.99	-0.33	0.596	12.66 ± 0.09	0.69	-0.45	0.226	12.86 ± 0.15	0.60	-0.23	0.591	13.08 ± 0.18	0.63	-0.51	0.213			
		F	11.18 ± 0.24				12.60 ± 0.18				12.83 ± 0.11				13.01 ± 0.11						
	**NQ**	H	11.33 ± 0.19				12.81 ± 0.15				13.05 ± 0.10				13.29 ± 0.11				1.35	-0.30	0.318
			**LAP 5**				**LAP 6**				**LAP 7**				**LAP 8**						
	**F**	H	13.08 ± 0.18	0.81	-0.26	0.549	13.26 ± 0.15	0.91	0.01	0.978	13.42 ± 0.14	1.00	-0.33	0.593	13.14 ± 0.37	1.06	-0.32	0.638			
		F	13.05 ± 0.21				13.26 ± 0.34				13.38 ± 0.39				13.10 ± 0.34						
	**NQ**	H	13.25 ± 0.09				13.36 ± 0.18				13.53 ± 0.17				13.26 ± 0.22						
			**LAP 1**				**LAP 2**				**LAP 3**				**LAP 4**				**Fixed effects (H-F)**		
**IN_5m**	**F**	H	2.80 ± 0.04	1.52	-0.53	0.494	2.91 ± 0.06	1.33	-1.03	0.227	2.99 ± 0.07	1.60	-2.13	0.057	3.00 ± 0.04	1.70	-0.41	0.662			
		F	2.78 ± 0.05				2.88 ± 0.04				2.93 ± 0.05				2.98 ± 0.07						
	**NQ**	H	2.83 ± 0.07				2.93 ± 0.03				3.01 ± 0.07				3.04 ± 0.06				1.59	-0.83	0.107
			**LAP 5**				**LAP 6**				**LAP 7**				**LAP 8**						
	**F**	H	3.04 ± 0.05	1.63	-0.66	0.464	3.03 ± 0.05	1.45	0.73	0.473	3.18 ± 0.09	1.91	-1.76	0.120							
		F	3.02 ± 0.07				3.06 ± 0.12				3.12 ± 0.11										
	**NQ**	H	3.09 ± 0.10				3.06 ± 0.04				3.15 ± 0.10										
			**LAP 1**				**LAP 2**				**LAP 3**				**LAP 4**				**Fixed effects (H-F)**		
**OUT 5m**	**F**	H					1.65 ± 0.05	1.40	0.45	0.620	1.71 ± 0.07	2.46	-0.58	0.757	1.66 ± 0.03	2.93	2.34	0.139			
		F					1.66 ± 0.06				1.70 ± 0.07				1.70 ± 0.06						
	**NQ**	H					1.72 ± 0.09				1.78 ± 0.07				1.72 ± 0.07				2.08	0.83	0.227
			**LAP 5**				**LAP 6**				**LAP 7**				**LAP 8**						
	**F**	H	1.75 ± 0.07	2.50	-1.01	0.587	1.70 ± 0.06	1.34	1.87	**0.005**	1.79 ± 0.07	2.43	1.51	0.225	1.75 ± 0.07	1.50	1.26	0.155			
		F	1.73 ± 0.07				1.73 ± 0.06				1.81 ± 0.10				1.77 ± 0.07						
	**NQ**	H	1.82 ± 0.07				1.75 ± 0.09				1.89 ± 0.10				1.83 ± 0.05						
			**LAP 1**				**LAP 2**				**LAP 3**				**LAP 4**				**Fixed effects (H-F)**		
**OUT 5_10m**	**F**	H					2.64 ± 0.06	0.73	-0.47	0.336	2.70 ± 0.03	1.11	0.82	0.195	2.73 ± 0.07	1.42	-0.73	0.451			
		F					2.63 ± 0.03				2.72 ± 0.03				2.71 ± 0.06						
	**NQ**	H					2.65 ± 0.03				2.73 ± 0.04				2.77 ± 0.07				1.21	-0.39	0.361
			**LAP 5**				**LAP 6**				**LAP 7**				**LAP 8**						
	**F**	H	2.75 ± 0.09	1.29	-0.54	0.498	2.75 ± 0.07	0.89	-0.54	0.473	2.80 ± 0.07	1.72	-1.53	0.078	2.83 ± 0.07	1.31	0.26	0.725			
		F	2.73 ± 0.05				2.74 ± 0.09				2.76 ± 0.12				2.84 ± 0.07						
	**NQ**	H	2.77 ± 0.06				2.75 ± 0.08				2.78 ± 0.10				2.85 ± 0.08						

			LAP 1				LAP 2				LAP 3				LAP 4				Fixed effects (H-F)		
**SR**	**F**	H	45.03 ± 3.59	1.91	0.73	0.558	42.16 ± 3.01	2.78	0.99	0.602	41.55 ± 2.21	1.95	1.67	0.123	41.78 ± 2.98	2.01	1.26	0.406			
		F	45.36 ± 4.16				42.58 ± 2.30				42.26 ± 2.01				42.32 ± 1.83						
	**NQ**	H	44.88 ± 1.09				43.10 ± 0.97				42.75 ± 1.53				41.70 ± 1.43				2.85	1.55	0.172
			**LAP 5**				**LAP 6**				**LAP 7**				**LAP 8**						
	**F**	H	42.35 ± 2.16	1.47	0.89	0.317	42.42 ± 2.09	3.91	1.68	0.523	43.19 ± 2.28	3.48	2.55	0.176	45.33 ± 2.64	2.88	2.64	0.070			
		F	42.73 ± 1.26				43.14 ± 2.62				44.32 ± 3.22				46.56 ± 3.54						
	**NQ**	H	43.34 ± 2.28				43.67 ± 1.42				44.25 ± 2.72				44.77 ± 2.12						
			**LAP 1**				**LAP 2**				**LAP 3**				**LAP 4**				**Fixed effects (H-F)**		
**SL**	**F**	H	2.59 ± 0.20	2.32	-0.64	0.637	2.68 ± 0.17	3.85	-0.44	0.860	2.64 ± 0.10	1.56	-1.94	**0.022**	2.58 ± 0.13	1.79	-1.10	0.386			
		F	2.57 ± 0.20				2.67 ± 0.09				2.59 ± 0.12				2.55 ± 0.09						
	**NQ**	H	2.56 ± 0.06				2.59 ± 0.05				2.53 ± 0.10				2.54 ± 0.12				2.83	-1.23	0.208
			**LAP 5**				**LAP 6**				**LAP 7**				**LAP 8**						
	**F**	H	2.54 ± 0.11	1.65	-0.47	0.675	2.51 ± 0.11	3.63	-1.53	0.523	2.46 ± 0.12	2.53	-2.07	0.152	2.32 ± 0.10	2.59	-1.68	0.230			
		F	2.53 ± 0.05				2.47 ± 0.13				2.41 ± 0.14				2.28 ± 0.13						
	**NQ**	H	2.45 ± 0.12				2.44 ± 0.09				2.38 ± 0.13				2.34 ± 0.11						
			**LAP 1**				**LAP 2**				**LAP 3**				**LAP 4**				**Fixed effects (H-F)**		
**CSS**	**F**	H	1.93 ± 0.04	1.63	-0.01	0.991	1.87 ± 0.04	1.68	0.82	0.510	1.82 ± 0.05	1.70	-0.26	0.784	1.79 ± 0.05	0.98	0.35	0.580			
		F	1.93 ± 0.03				1.89 ± 0.07				1.82 ± 0.03				1.79 ± 0.02						
	**NQ**	H	1.91 ± 0.06				1.86 ± 0.05				1.80 ± 0.03				1.76 ± 0.03				1.67	0.34	0.385
			**LAP 5**				**LAP 6**				**LAP 7**				**LAP 8**						
	**F**	H	1.79 ± 0.04	1.30	0.56	0.471	1.77 ± 0.04	1.44	0.06	0.943	1.76 ± 0.03	1.07	0.43	0.565	1.75 ± 0.05	1.34	0.83	0.332			
		F	1.80 ± 0.03				1.77 ± 0.02				1.77 ± 0.06				1.76 ± 0.04						
	**NQ**	H	1.76 ± 0.05				1.77 ± 0.03				1.75 ± 0.01				1.75 ± 0.36						

Table S6. Inter-rounds variability in performance and fixed effects obtained for the Finalists (n = 8) and the Non-Qualified swimmers (n = 8) in the 200m breaststroke.

			LAP 1				LAP 2				LAP 3				LAP 4				Fixed effects (H-F)		
			Mean	CV	Δ%	p	Mean	CV	Δ%	p	Mean	CV	Δ%	p	Mean	CV	Δ%	p	CV	Δ%	p
Split	F	H	12.88 ± 0.27	1.08	-0.94	0.117	15.51 ± 0.19	1.01	-1.14	0.035	15.73 ± 0.22	1.07	-1.10	0.044	15.90 ± 0.12	0.48	-0.68	0.008			
		F	12.76 ± 0.30				15.33 ± 0.12				15.56 ± 0.13				15.79 ± 0.12						
	NQ	H	13.11 ± 0.22				15.70 ± 0.17				15.95 ± 0.14				16.20 ± 0.17				1.52	-0.61	0.009
			LAP 5				LAP 6				LAP 7				LAP 8						
	F	H	15.85 ± 0.18	0.70	0.17	0.650	16.02 ± 0.19	0.73	-0.21	0.592	16.06 ± 0.27	0.64	-0.12	0.794	16.11 ± 0.28	1.09	-0.84	0.158			
		F	15.88 ± 0.18				15.99 ± 0.18				16.04 ± 0.29				15.98 ± 0.34						
	NQ	H	16.23 ± 0.27				16.43 ± 0.15				16.49 ± 0.27				16.67 ± 0.16						
			LAP 1				LAP 2				LAP 3				LAP 4				Fixed effects (H-F)		
IN_5m	F	H	2.94 ± 0.11	2.47	1.25	0.358	3.09 ± 0.10	1.09	-0.56	0.403	3.14 ± 0.08	1.41	-0.88	0.312	3.21 ± 0.09	1.27	-0.70	0.368			
		F	2.98 ± 0.11				3.07 ± 0.05				3.11 ± 0.07				3.19 ± 0.07						
	NQ	H	2.94 ± 0.05				3.04 ± 0.09				3.11 ± 0.07				3.15 ± 0.06				1.82	0.43	0.392
			LAP 5				LAP 6				LAP 7				LAP 8						
	F	H	3.10 ± 0.09	2.80	3.42	0.009	3.23 ± 0.07	2.15	-1.01	0.417	3.21 ± 0.09	1.53	1.53	0.053							
		F	3.21 ± 0.08				3.20 ± 0.09				3.26 ± 0.09										
	NQ	H	3.18 ± 0.11				3.21 ± 0.08				3.27 ± 0.08										
			LAP 1				LAP 2				LAP 3				LAP 4				Fixed effects (H-F)		
OUT 5m	F	H					2.52 ± 0.07	2.56	-3.27	0.010	2.57 ± 0.06	2.37	-0.97	0.499	2.58 ± 0.07	1.58	-1.87	0.030			
		F					2.44 ± 0.08				2.55 ± 0.08				2.53 ± 0.06						
	NQ	H					2.59 ± 0.09				2.68 ± 0.10				2.70 ± 0.12				2.19	-1.51	0.006
			LAP 5				LAP 6				LAP 7				LAP 8						
	F	H	2.66 ± 0.06	2.78	1.02	0.635	2.63 ± 0.06	1.91	-2.33	0.022	2.69 ± 0.10	1.84	-0.37	0.724	2.68 ± 0.09	2.32	-2.77	0.023			
		F	2.68 ± 0.16				2.57 ± 0.07				2.68 ± 0.07				2.61 ± 0.06						
	NQ	H	2.78 ± 0.15				2.75 ± 0.10				2.81 ± 0.15				2.79 ± 0.15						
			LAP 1				LAP 2				LAP 3				LAP 4				Fixed effects (H-F)		
OUT 5_10m	F	H					3.12 ± 0.15	3.11	-4.25	0.050	3.00 ± 0.25	4.46	-0.83	0.799	3.15 ± 0.16	1.45	-0.31	0.740			
		F					2.99 ± 0.17				2.97 ± 0.17				3.14 ± 0.16						
	NQ	H					3.17 ± 0.17				3.19 ± 0.22				3.30 ± 0.11				3.03	-1.14	0.362
			LAP 5				LAP 6				LAP 7				LAP 8						
	F	H	3.21 ± 0.13	4.61	-2.37	0.425	3.21 ± 0.13	2.89	1.22	0.458	3.20 ± 0.23	3.45	-0.54	0.790	3.26 ± 0.15	1.22	-0.92	0.211			
		F	3.05 ± 0.23				3.25 ± 0.11				3.18 ± 0.13				3.23 ± 0.19						
	NQ	H	3.28 ± 0.10				3.33 ± 0.11				3.40 ± 0.09				3.48 ± 0.06						

			LAP 1				LAP 2				LAP 3				LAP 4				Fixed effects (H-F)		
**SR**	**F**	H	36.53 ± 6.84	12.56	-0.44	0.955	32.44 ± 3.16	4.28	3.52	0.121	31.41 ± 3.31	4.21	4.55	0.069	32.35 ± 3.44	3.56	-0.31	0.891			
		F	36.37 ± 6.31				33.63 ± 3.15				32.90 ± 2.80				32.25 ± 2.18						
	**NQ**	H	37.30 ± 3.57				35.08 ± 4.32				34.26 ± 3.01				33.78 ± 4.22				6.78	2.13	0.281
			**LAP 5**				**LAP 6**				**LAP 7**				**LAP 8**						
	**F**	H	34.89 ± 2.08	4.62	-1.96	0.481	35.42 ± 4.54	4.41	-1.07	0.698	38.07 ± 4.11	4.27	3.47	0.215	43.67 ± 5.60	8.54	9.31	0.063			
		F	34.21 ± 3.75				35.04 ± 3.27				39.44 ± 3.65				48.15 ± 3.81						
	**NQ**	H	35.87 ± 4.33				36.55 ± 3.50				40.44 ± 3.39				44.04 ± 3.60						
			**LAP 1**				**LAP 2**				**LAP 3**				**LAP 4**				**Fixed effects (H-F)**		
**SL**	**F**	H	2.49 ± 0.37	10.87	4.47	0.532	2.72 ± 0.22	2.64	-2.06	0.287	2.81 ± 0.26	4.25	-3.76	0.160	2.71 ± 0.25	4.42	0.37	0.893			
		F	2.61 ± 0.40				2.67 ± 0.20				2.71 ± 0.21				2.72 ± 0.18						
	**NQ**	H	2.43 ± 0.18				2.55 ± 0.23				2.57 ± 0.17				2.57 ± 0.29				6.18	-1.60	0.51
			**LAP 5**				**LAP 6**				**LAP 7**				**LAP 8**						
	**F**	H	2.50 ± 0.17	4.73	3.43	0.215	2.49 ± 0.31	4.03	0.01	0.995	2.30 ± 0.22	4.38	-4.35	0.139	2.05 ± 0.26	7.73	-10.97	0.052			
		F	2.58 ± 0.27				2.49 ± 0.22				2.21 ± 0.18				1.85 ± 0.13						
	**NQ**	H	2.42 ± 0.22				2.36 ± 0.21				2.14 ± 0.17				1.97 ± 0.15						
			**LAP 1**				**LAP 2**				**LAP 3**				**LAP 4**				**Fixed effects (H-F)**		
**CSS**	**F**	H	1.48 ± 0.08	3.32	4.08	0.062	1.46 ± 0.04	2.58	1.64	0.326	1.46 ± 0.05	1.93	1.21	0.298	1.45 ± 0.06	2.51	0.48	0.748			
		F	1.55 ± 0.06				1.48 ± 0.06				1.47 ± 0.04				1.45 ± 0.03						
	**NQ**	H	1.50 ± 0.04				1.47 ± 0.04				1.46 ± 0.04				1.43 ± 0.03				2.03	0.96	0.034
			**LAP 5**				**LAP 6**				**LAP 7**				**LAP 8**						
	**F**	H	1.44 ± 0.04	1.05	0.87	0.130	1.45 ± 0.05	1.60	-0.43	0.652	1.45 ± 0.05	1.25	-0.50	0.508	1.47 ± 0.02	1.11	0.32	0.662			
		F	1.46 ± 0.30				1.44 ± 0.03				1.44 ± 0.04				1.48 ± 0.03						
	**NQ**	H	1.43 ± 0.03				1.42 ± 0.03				1.43 ± 0.03				1.44 ± 0.01						

Table S7. Inter-rounds variability in performance and fixed effects obtained for the Finalists (F; n = 8) and the Non-Qualified swimmers (NQ; n = 8) in the 200m backstroke.

			LAP 1				LAP 2				LAP 3				LAP 4				Fixed effects (H-F)		
			Mean	CV	Δ%	p	Mean	CV	Δ%	p	Mean	CV	Δ%	p	Mean	CV	Δ%	p	CV	Δ%	p
**Split**	**F**	H	12.31 ± 0.19	0.39	0.34	0.151	13.57 ± 0.17	0.52	-1.30	1.0	13.84 ± 0.15	1.09	0.89	0.160	14.17 ± 0.17	1.02	0.10	0.861			
		F	12.35 ± 0.21				13.57 ± 0.18				13.96 ± 0.24				14.18 ± 0.26						
	**NQ**	H	12.42 ± 0.18				13.66 ± 0.18				14.09 ± 0.16				14.39 ± 0.12				1.41	-0.06	0.814
			**LAP 5**				**LAP 6**				**LAP 7**				**LAP 8**						
	**F**	H	14.11 ± 0.07	0.91	0.75	0.169	14.35 ± 0.15	1.00	0.08	0.903	14.37 ± 0.28	0.84	-0.73	0.140	14.26 ± 0.41	2.01	-1.94	0.115			
		F	14.22 ± 0.25				14.36 ± 0.34				14.27 ± 0.25				13.98 ± 0.38						
	**NQ**	H	14.48 ± 0.10				14.73 ± 0.04				14.74 ± 0.18				14.62 ± 0.34						
			**LAP 1**				**LAP 2**				**LAP 3**				**LAP 4**				**Fixed effects (H-F)**		
**IN_5m**	**F**	H	3.09 ± 0.23	3.72	-1.22	0.652	3.23 ± 0.05	1.26	0.99	0.169	3.25 ± 0.09	1.83	-0.15	0.904	3.30 ± 0.07	1.76	0.52	0.609			
		F	3.06 ± 0.08				3.26 ± 0.05				3.24 ± 0.09				3.31 ± 0.10						
	**NQ**	H	2.98 ± 0.05				3.22 ± 0.08				3.23 ± 0.08				3.30 ± 0.07				1.91	-0.14	0.796
			**LAP 5**				**LAP 6**				**LAP 7**				**LAP 8**						
	**F**	H	3.33 ± 0.05	1.68	1.18	0.219	3.34 ± 0.09	1.64	-0.52	0.626	3.43 ± 0.14	1.51	-1.85	0.020							
		F	3.37 ± 0.11				3.32 ± 0.10				3.37 ± 0.12										
	**NQ**	H	3.39 ± 0.05				3.35 ± 0.06				3.42 ± 0.04										
			**LAP 1**				**LAP 2**				**LAP 3**				**LAP 4**				**Fixed effects (H-F)**		
**OUT 5m**	**F**	H					1.73 ± 0.16	4.96	-3.50	0.354	1.81 ± 0.06	2.77	-1.39	0.437	1.75 ± 0.10	3.10	0.42	0.824			
		F					1.67 ± 0.08				1.78 ± 0.10				1.75 ± 0.09						
	**NQ**	H					1.70 ± 0.04				1.70 ± 0.15				1.73 ± 0.13				2.76	-0.41	0.663
			**LAP 5**				**LAP 6**				**LAP 7**				**LAP 8**						
	**F**	H	1.83 ± 0.06	2.19	2.01	0.130	1.76 ± 0.10	1.80	-1.25	1.0	1.85 ± 0.06	2.08	-0.81	0.572	1.83 ± 0.09	2.40	0.40	0.782			
		F	1.87 ± 0.05				1.76 ± 0.06				1.83 ± 0.07				1.84 ± 0.06						
	**NQ**	H	1.78 ± 0.08				**1.79 ± 0.06**				**1.87 ± 0.09**				**1.83 ± 0.09**						
			**LAP 1**				**LAP 2**				**LAP 3**				**LAP 4**				**Fixed effects (H-F)**		
**OUT 5_10m**	**F**	H					2.64 ± 0.24	4.25	3.24	0.336	2.71 ± 0.10	3.41	0.73	0.710	2.82 ± 0.05	1.93	0.61	0.628			
		F					2.73 ± 0.12				2.73 ± 0.15				2.84 ± 0.10						
	**NQ**	H					2.79 ± 0.12				2.88 ± 0.12				3.01 ± 0.12				2.4	0.78	0.414
			**LAP 5**				**LAP 6**				**LAP 7**				**LAP 8**						
	**F**	H	2.76 ± 0.12	1.53	0.89	0.271	2.92 ± 0.09	1.65	0.76	0.467	2.82 ± 0.18	1.11	0.87	0.401	2.99 ± 0.11	2.93	-1.61	0.333			
		F	2.79 ± 0.17				2.94 ± 0.10				2.84 ± 0.16				2.94 ± 0.11						
	**NQ**	H	2.90 ± 0.12				3.02 ± 0.12				2.94 ± 0.12				3.16 ± 0.12						

			**LAP 1**				**LAP 2**				**LAP 3**				**LAP 4**				**Fixed effects (H-F)**		
**SR**	**F**	H	45.78 ± 3.50	2.16	0.03	0.979	42.09 ± 3.20	2.83	-2.28	0.157	40.44 ± 3.76	3.01	-0.25	0.897	40.69 ± 3.19	2.31	-1.37	0.438			
		F	45.80 ± 3.64				41.15 ± 2.99				40.34 ± 2.50				40.13 ± 2.47						
	**NQ**	H	46.93 ± 2.85				42.44 ± 2.39				40.95 ± 1.87				40.41 ± 1.87				3.16	0.09	0.909
			**LAP 5**				**LAP 6**				**LAP 7**				**LAP 8**						
	**F**	H	41.11 ± 2.68	2.05	-1.27	0.372	41.58 ± 3.07	4.30	-0.56	0.852	41.97 ± 3.65	2.98	2.05	0.349	42.50 ± 2.85	3.25	4.40	0.003			
		F	40.59 ± 2.32				41.35 ± 2.29				42.85 ± 1.87				44.46 ± 2.23						
	**NQ**	H	40.50 ± 2.18				40.14 ± 1.85				41.44 ± 1.79				42.74 ± 1.46						
			**LAP 1**				**LAP 2**				**LAP 3**				**LAP 4**				**Fixed effects (H-F)**		
**SL**	**F**	H	2.36 ± 0.32	4.20	-4.15	0.298	2.46 ± 0.18	2.72	2.65	0.103	2.48 ± 0.21	2.70	-1.87	0.248	2.36 ± 0.15	1.99	2.28	0.055			
		F	2.27 ± 0.18				2.52 ± 0.18				2.43 ± 0.14				2.42 ± 0.16						
	**NQ**	H	2.23 ± 0.12				2.42 ± 0.13				2.39 ± 0.10				2.32 ± 0.14				3.13	-0.68	0.558
			**LAP 5**				**LAP 6**				**LAP 7**				**LAP 8**						
	**F**	H	2.40 ± 0.17	1.93	0.41	0.699	2.28 ± 0.15	3.34	0.06	0.976	2.35 ± 0.23	3.22	-2.51	0.201	2.25 ± 0.17	2.85	-2.30	0.298			
		F	2.41 ± 0.12				2.28 ± 0.11				2.29 ± 0.14				2.20 ± 0.11						
	**NQ**	H	2.37 ± 0.12				2.28 ± 0.09				2.29 ± 0.11				2.19 ± 0.09						
			**LAP 1**				**LAP 2**				**LAP 3**				**LAP 4**				**Fixed effects (H-F)**		
**CSS**	**F**	H	1.79 ± 0.15	4.33	-3.95	0.203	1.71 ± 0.03	1.18	0.46	0.525	1.66 ± 0.05	1.53	-1.77	0.060	1.59 ± 0.03	1.53	1.02	0.251			
		F	1.72 ± 0.03				1.72 ± 0.02				1.63 ± 0.04				1.61 ± 0.02						
	**NQ**	H	1.74 ± 0.03				1.71 ± 0.05				1.63 ± 0.02				1.56 ± 0.03				2.37	-0.33	0.539
			**LAP 5**				**LAP 6**				**LAP 7**				**LAP 8**						
	**F**	H	1.64 ± 0.03	1.15	-0.70	0.273	1.57 ± 0.03	2.33	-0.26	0.831	1.63 ± 0.04	2.13	0.12	0.924	1.59 ± 0.05	2.47	2.41	0.064			
		F	1.63 ± 0.04				1.57 ± 0.05				1.63 ± 0.06				1.63 ± 0.04						
	**NQ**	H	1.59 ± 0.03				1.52 ± 0.03				1.58 ± 0.02				1.55 ± 0.04						

Table S8. Inter-rounds variability in performance and fixed effects obtained for the Finalists (F; n = 8) and the Non-Qualified swimmers (NQ; n = 8) in the 200m butterfly.

			LAP 1				LAP 2				LAP 3				LAP 4				Fixed effects (H-F)		
			**Mean**	**CV**	**Δ%**	**p**	**Mean**	**CV**	**Δ%**	**p**	**Mean**	**CV**	**Δ%**	**p**	**Mean**	**CV**	**Δ%**	**p**	**CV**	**Δ%**	**p**
**Split**	**F**	H	11.43 ± 0.19	0.99	-1.23	0.005	14.11 ± 0.16	1.60	-2.28	0.002	14.24 ± 0.27	0.74	-0.47	0.282	14.43 ± 0.29	0.75	-0.61	0.161			
		F	11.29 ± 0.15				13.79 ± 0.14				14.17 ± 0.22				14.34 ± 0.24						
	**NQ**	H	11.47 ± 0.12				13.97 ± 0.24				14.48 ± 0.13				14.70 ± 0.30				1.92	-0.76	0.051
			**LAP 5**				**LAP 6**				**LAP 7**				**LAP 8**						
	**F**	H	14.45 ± 0.22	0.58	-0.39	0.302	14.64 ± 0.12	0.84	0.47	0.351	14.73 ± 0.13	2.02	-0.34	0.794	15.01 ± 0.37	1.78	-1.24	0.263			
		F	14.39 ± 0.26				14.71 ± 0.22				14.68 ± 0.55				14.82 ± 0.30						
	**NQ**	H	14.69 ± 0.19				14.95 ± 0.15				14.94 ± 0.16				15.11 ± 0.33						
			**LAP 1**				**LAP 2**				**LAP 3**				**LAP 4**				**Fixed effects (H-F)**		
**IN_5m**	**F**	H	2.57 ± 0.07	3.40	-0.48	0.790	2.81 ± 0.07	3.02	-2.97	0.103	2.78 ± 0.10	1.79	0.35	0.740	2.82 ± 0.09	1.74	0.01	1.0			
		F	2.56 ± 0.11				2.73 ± 0.16				2.79 ± 0.08				2.82 ± 0.13						
	**NQ**	H	2.60 ± 0.12				2.77 ± 0.13				2.88 ± 0.10				2.86 ± 0.12				2.76	0.04	0.930
			**LAP 5**				**LAP 6**				**LAP 7**				**LAP 8**						
	**F**	H	2.89 ± 0.09	1.04	0.01	0.892	2.84 ± 0.12	4.35	3.14	0.169	3.02 ± 0.12	3.99	0.16	0.951							
		F	2.89 ± 0.11				2.94 ± 0.14				3.03 ± 0.20										
	**NQ**	H	2.91 ± 0.08				2.97 ± 0.10				3.03 ± 0.11										
			**LAP 1**				**LAP 2**				**LAP 3**				**LAP 4**				**Fixed effects (H-F)**		
**OUT 5m**	**F**	H					2.65 ± 0.09	3.38	-2.21	0.311	2.73 ± 0.09	2.52	-1.15	0.463	2.69 ± 0.09	2.16	0.46	0.733			
		F					2.59 ± 0.15				2.70 ± 0.19				2.70 ± 0.12						
	**NQ**	H					2.56 ± 0.13				2.69 ± 0.09				2.67 ± 0.12				2.82	-2.19	0.016
			**LAP 5**				**LAP 6**				**LAP 7**				**LAP 8**						
	**F**	H	2.84 ± 0.09	3.05	-3.64	0.047	2.77 ± 0.14	1.96	-1.92	0.107	2.91 ± 0.06	3.89	-5.53	0.002	2.76 ± 0.12	2.77	-1.37	0.350			
		F	2.74 ± 0.12				2.72 ± 0.12				2.75 ± 0.15				2.72 ± 0.09						
	**NQ**	H	2.76 ± 0.08				2.70 ± 0.12				2.80 ± 0.10				2.76 ± 0.12						
			**LAP 1**				**LAP 2**				**LAP 3**				**LAP 4**				**Fixed effects (H-F)**		
**OUT 5_10m**	**F**	H					2.80 ± 0.08	1.59	-1.53	0.095	2.81 ± 0.19	2.21	-0.98	0.431	2.80 ± 0.12	1.55	1.14	0.325			
		F					2.76 ± 0.10				2.78 ± 0.13				2.83 ± 0.13						
	**NQ**	H					2.79 ± 0.08				2.93 ± 0.06				2.92 ± 0.07				2.24	-0.48	0.536
			**LAP 5**				**LAP 6**				**LAP 7**				**LAP 8**						
	**F**	H	2.79 ± 0.14	1.71	-0.63	0.582	2.78 ± 0.09	2.27	1.59	0.300	2.79 ± 0.10	2.78	0.17	0.917	2.93 ± 0.10	3.60	-3.16	0.079			
		F	2.77 ± 0.17				2.82 ± 0.14				2.80 ± 0.15				2.84 ± 0.09						
	**NQ**	H	2.92 ± 0.07				2.91 ± 0.05				2.91 ± 0.10				2.95 ± 0.17						

			LAP 1				LAP 2				LAP 3				LAP 4				Fixed effects (H-F)		
SR	F	H	50.56 ± 1.58	2.61	2.53	0.105	48.20 ± 2.44	1.28	0.61	0.545	48.00 ± 2.39	1.14	0.85	0.236	48.50 ± 2.50	1.58	-0.19	0.823			
		F	51.88 ± 3.11				48.50 ± 2.55				48.42 ± 2.68				48.40 ± 2.48						
	NQ	H	51.69 ± 2.21				48.78 ± 3.25				48.21 ± 3.93				48.10 ± 3.92				1.96	0.99	0.087
			LAP 5				LAP 6				LAP 7				LAP 8						
	F	H	48.96 ± 2.11	1.59	-0.61	0.482	48.92 ± 1.50	1.60	0.28	0.753	48.86 ± 2.05	2.94	3.34	0.022	50.99 ± 1.56	1.37	1.12	0.315			
		F	48.66 ± 2.10				49.05 ± 2.10				50.55 ± 2.76				51.57 ± 2.10						
	NQ	H	49.37 ± 3.96				48.93 ± 2.89				51.07 ± 2.63				51.81 ± 2.39						
			LAP 1				LAP 2				LAP 3				LAP 4				Fixed effects (H-F)		
SL	F	H	2.13 ± 0.09	2.98	-1.48	0.414	2.17 ± 0.15	2.51	2.47	0.098	2.10 ± 0.11	1.33	1.26	0.202	2.07 ± 0.07	2.05	-0.21	0.870			
		F	2.10 ± 0.13				2.22 ± 0.15				2.12 ± 0.13				2.07 ± 0.12						
	NQ	H	2.05 ± 0.09				2.22 ± 0.20				2.08 ± 0.14				2.04 ± 0.21				2.59	-0.46	0.575
			LAP 5				LAP 6				LAP 7				LAP 8						
	F	H	2.04 ± 0.10	1.61	1.47	0.068	2.01 ± 0.06	2.09	-2.24	0.066	2.04 ± 0.08	3.42	-4.44	0.004	1.89 ± 0.06	1.53	-0.51	0.616			
		F	2.07 ± 0.09				1.97 ± 0.07				1.95 ± 0.13				1.88 ± 0.05						
	NQ	H	1.97 ± 0.10				1.96 ± 0.12				1.90 ± 0.12				1.85 ± 0.07						
			LAP 1				LAP 2				LAP 3				LAP 4				Fixed effects (H-F)		
CSS	F	H	1.80 ± 0.07	2.94	1.01	0.541	1.74 ± 0.07	2.51	3.06	0.026	1.67 ± 0.05	2.06	2.05	0.068	1.67 ± 0.04	2.12	-0.50	0.716			
		F	1.81 ± 0.11				1.79 ± 0.07				1.71 ± 0.05				1.66 ± 0.04						
	NQ	H	1.76 ± 0.07				1.79 ± 0.08				1.66 ± 0.05				1.63 ± 0.06				2.51	0.50	0.369
			LAP 5				LAP 6				LAP 7				LAP 8						
	F	H	1.66 ± 0.04	1.31	0.89	0.392	1.64 ± 0.02	2.40	-1.96	0.125	1.66 ± 0.04	2.60	-1.05	0.491	1.61 ± 0.05	1.55	0.56	0.633			
		F	1.68 ± 0.05				1.60 ± 0.05				1.64 ± 0.08				1.62 ± 0.03						
	NQ	H	1.62 ± 0.05				1.59 ± 0.03				1.61 ± 0.03				1.59 ± 0.03						
